# Age-of-onset information helps identify 76 genetic variants associated with allergic disease

**DOI:** 10.1371/journal.pgen.1008725

**Published:** 2020-06-30

**Authors:** Manuel A. R. Ferreira, Judith M. Vonk, Hansjörg Baurecht, Ingo Marenholz, Chao Tian, Joshua D. Hoffman, Quinta Helmer, Annika Tillander, Vilhelmina Ullemar, Yi Lu, Sarah Grosche, Franz Rüschendorf, Raquel Granell, Ben M. Brumpton, Lars G. Fritsche, Laxmi Bhatta, Maiken E. Gabrielsen, Jonas B. Nielsen, Wei Zhou, Kristian Hveem, Arnulf Langhammer, Oddgeir L. Holmen, Mari Løset, Gonçalo R. Abecasis, Cristen J. Willer, Nima C. Emami, Taylor B. Cavazos, John S. Witte, Agnieszka Szwajda, the 23andMe Research Team, collaborators of the SHARE study, David A. Hinds, Norbert Hübner, Stephan Weidinger, Patrik KE Magnusson, Eric Jorgenson, Robert Karlsson, Lavinia Paternoster, Dorret I. Boomsma, Catarina Almqvist, Young-Ae Lee, Gerard H. Koppelman

**Affiliations:** 1 Genetics and Computational Biology, QIMR Berghofer Medical Research Institute, Brisbane, Australia; 2 University of Groningen, University Medical Center Groningen, Epidemiology, Groningen Research Institute for Asthma and COPD, Groningen, the Netherlands; 3 Department of Dermatology, Allergology and Venereology, University Hospital Schleswig-Holstein, Campus Kiel, Kiel, Germany; 4 Department of Epidemiology and Preventive Medicine, University of Regensburg, Regensburg, Germany; 5 Max Delbrück Center (MDC) for Molecular Medicine, Berlin, Germany; 6 Clinic for Pediatric Allergy, Experimental and Clinical Research Center of Charité Universitätsmedizin Berlin and Max Delbrück Center, Berlin, Germany; 7 23andMe, Inc., Mountain View, California, United States of America; 8 Department of Epidemiology and Biostatistics, University of California San Francisco, San Francisco, California, United States of America; 9 Department Biological Psychology, Netherlands Twin Register, Vrije University, Amsterdam, The Netherlands; 10 Department of Medical Epidemiology and Biostatistics and the Swedish Twin Registry, Karolinska Institutet, Stockholm, Sweden; 11 CeMM Research Center for Molecular Medicine of the Austrian Academy of Sciences, Vienna, Austria; 12 MRC Integrative Epidemiology Unit, Population Health Sciences, University of Bristol, Bristol, United Kingdom; 13 K.G. Jebsen Center for Genetic Epidemiology, Department of Public Health and Nursing, NTNU, Norwegian University of Science and Technology, Trondheim, Norway; 14 Department of Thoracic Medicine, St. Olavs Hospital, Trondheim University Hospital, Trondheim, Norway; 15 Department of Biostatistics and Center for Statistical Genetics, University of Michigan, Ann Arbor, Michigan, United States of America; 16 Department of Human Genetics, University of Michigan, Ann Arbor, Michigan, United States of America; 17 Department of Internal Medicine, University of Michigan, Ann Arbor, Michigan, United States of America; 18 The HUNT Research Centre, Department of Public Health and Nursing, NTNU, Norwegian University of Science and Technology, Trondheim, Norway; 19 Department of Dermatology, St. Olavs Hospital, Trondheim University Hospital, Trondheim, Norway; 20 Program in Biological and Medical Informatics, University of California, San Francisco, San Francisco, California, United States of America; 21 Department of Epidemiology and Biostatistics, University of California, San Francisco, San Francisco, California, United States of America; 22 Institute for Human Genetics, University of California, San Francisco, San Francisco, California, United States of America; 23 Department of Urology, Helen Diller Family Comprehensive Cancer Center, University of California, San Francisco, California, United States of America; 24 Department of Medical Epidemiology and Biostatistics, Karolinska Institutet, Stockholm, Sweden; 25 Division of Research, Kaiser Permanente Northern California, Oakland, California, United States of America; 26 Pediatric Allergy and Pulmonology Unit at Astrid Lindgren Children’s Hospital, Karolinska University Hospital, Stockholm, Sweden; 27 University of Groningen, University Medical Center Groningen, Beatrix Children’s Hospital, Pediatric Pulmonology and Pediatric Allergology, and University of Groningen, University Medical Center Groningen, Groningen Research Institute for Asthma and COPD, Groningen, the Netherlands; INSERM, FRANCE

## Abstract

Risk factors that contribute to inter-individual differences in the age-of-onset of allergic diseases are poorly understood. The aim of this study was to identify genetic risk variants associated with the age at which symptoms of allergic disease first develop, considering information from asthma, hay fever and eczema. Self-reported age-of-onset information was available for 117,130 genotyped individuals of European ancestry from the UK Biobank study. For each individual, we identified the earliest age at which asthma, hay fever and/or eczema was first diagnosed and performed a genome-wide association study (GWAS) of this combined age-of-onset phenotype. We identified 50 variants with a significant independent association (*P*<3x10^-8^) with age-of-onset. Forty-five variants had comparable effects on the onset of the three individual diseases and 38 were also associated with allergic disease case-control status in an independent study (*n* = 222,484). We observed a strong negative genetic correlation between age-of-onset and case-control status of allergic disease (*r*_*g*_ = -0.63, *P* = 4.5x10^-61^), indicating that cases with early disease onset have a greater burden of allergy risk alleles than those with late disease onset. Subsequently, a multivariate GWAS of age-of-onset and case-control status identified a further 26 associations that were missed by the univariate analyses of age-of-onset or case-control status only. Collectively, of the 76 variants identified, 18 represent novel associations for allergic disease. We identified 81 likely target genes of the 76 associated variants based on information from expression quantitative trait loci (eQTL) and non-synonymous variants, of which we highlight *ADAM15*, *FOSL2*, *TRIM8*, *BMPR2*, *CD200R1*, *PRKCQ*, *NOD2*, *SMAD4*, *ABCA7* and *UBE2L3*. Our results support the notion that early and late onset allergic disease have partly distinct genetic architectures, potentially explaining known differences in pathophysiology between individuals.

## Author summary

So far, genetic studies of allergic disease have investigated the presence of the disease rather than the age at which the first allergic symptoms develop. We aimed to identify genetic risk variants associated with the age at which symptoms of allergic disease first develop, considering information from asthma, hay fever and eczema by examining 117,130 genotyped individuals of European ancestry from the UK Biobank study. We identified 50 variants with a significant independent association (*P*<3x10^-8^) with age-of-onset. Forty-five variants had comparable effects on the onset of the three individual diseases and 38 were also associated with allergic disease case-control status in an independent study (*n* = 222,484). We then performed a multivariate GWAS of age-of-onset and case-control status identified a further 26 associations that were missed by the univariate analyses of age-of-onset or case-control status only. 18 of 76 variants identified represent novel associations for allergic disease. We identified 81 likely target genes of the 76 genetic variants, including *ADAM15*, *FOSL2*, *TRIM8*, *BMPR2*, *CD200R1*, *PRKCQ*, *NOD2*, *SMAD4*, *ABCA7* and *UBE2L3*. Our results support the notion that early and late onset allergic disease have partly distinct genetic architectures, potentially explaining known differences in pathophysiology between individuals.

 

## Introduction

In the last 10 years, at least 45 genome-wide association studies (GWAS) of allergic disease susceptibility were published: 25 for asthma (reviewed in [1]), three for hay fever (or allergic rhinitis) [2–4], eight for eczema (or atopic dermatitis) [5–12], four for food allergy [13–16] and six for allergy-related traits, namely atopic march [17], asthma with hay fever [18], allergies [19], allergic sensitization [2,20,21] and a combined asthma, hay fever and eczema phenotype [22]. Genetic risk variants identified in these studies provide a foundation to help us better understand why and how allergic disease develops in susceptible individuals.

One twin study has previously indicated that the timing of asthma onset may be under genetic control [23]. In the first genome wide association study for asthma published in 2007, it was reported that the *ORMDL3/GSDMA* locus at chromosome 17q12 was specifically associated with childhood onset asthma[24]. This observation was subsequently confirmed, showing strong associations of this locus with childhood-onset asthma, potentially interacting with passive cigarette smoke exposure in early childhood [25] or as childhood onset asthma defined as asthma developing before 16 years of age) but not later onset asthma in the GABRIEL consortium [26] In subsequent stratified analyses in a multinational study, it was reported that the association of the 17q risk SNP rs7216389-T was confined to cases with early onset of asthma, particularly in early childhood (age: 0–5 years) and adolescence (age: 14–17 years), but a weaker association was observed for onset between 6 and 13 years of age, whereas no association was observed for adult-onset asthma [27]. This shows that defining cut-offs for age at onset of asthma is difficult, and that other approaches such as using a continuous age at onset might be beneficial.

To our knowledge, only three studies have reported genetic variants that associate with the age at which allergic disease symptoms first develop. Forno et al. [28] studied asthma age-of-onset in 573 children and identified two variants that had a genome-wide significant association after combining the discovery and replication (*n* = 931) cohorts: rs9815663 near the *CRBN* gene on chromosome 3p26, and rs7927044 near *ETS1* on 11q24. In a more recent GWAS of 5,462 cases with asthma, Sarnowski et al. [29] identified five variants associated with age-of-onset, located in/near: *CYLD* on 16q12 (rs1861760), *IL1RL1* on 2q12 (rs10208293), *HLA-DQA1* on 6p21 (rs9272346), *IL33* on 9p24 (rs928413) and *GSDMA* on 17q12 (rs9901146). The latter four variants were previously reported to be associated with allergic disease susceptibility as well. Lastly, Ferreira et al. [22] reported that 26 of 136 variants associated with allergic disease risk were also associated with the age at which allergic symptoms first developed (*n* = 35,972). Amongst these were five variants for which the association with age-of-onset was genome-wide significant: rs61816761 in the *FLG* gene and rs12123821 near *HRNR*, both on chromosome 1q21; rs921650 in *GSDMB* on 17q12; rs10865050 in *IL18R1* on 2q12; and rs7936323 near *LRRC32* on 11q13. Two of the variants reported in Ferreira et al. (rs10865050 and rs921650) were in linkage disequilibrium (LD) with variants reported in Sarnowski et al., and so are unlikely to represent independent associations. Therefore, collectively across these three studies, 12 variants (2+5+5, including 10 in low LD with each other) were reported to associate with age-of-onset of allergic disease at the genome-wide significance level. Of interest, the joint association between age-at-onset and disease susceptibility at some of these loci [29] suggests that both phenotypes are genetically correlated, and so that combining information from both may improve power to identify variants that influence the aetiology of allergic disease.

The main aim of this study was to identify novel loci that contribute to inter-individual variability in the age at which allergic symptoms first develop, considering information from the three most common allergic diseases: asthma, hay fever and eczema. Rather than study the age-of-onset of each disease separately, we adopted the multi-disease phenotype approach that we used recently to identify risk variants that are shared across different allergic diseases [22]. Specifically, we determined the earliest age at which asthma and/or hay fever and/or eczema first developed and then tested this single combined age-of-onset of allergic disease phenotype in a GWAS. In addition, we also tested if variants associated with disease age-of-onset were also associated with disease risk, as noted by Sarnowski et al. [29]. Lastly, we used multivariate association analysis to identify variants jointly associated with allergic disease age-of-onset and case-control status, which were missed by analyzing each phenotype alone.

## Results

### Genetic variants associated with the age-of-onset of allergic disease

Our study population consisted of *n* = 117,130 participants from the UK Biobank study **(S2 Table),** who had a mean age of 55.5 years (range 38–72 years), with a mean (median) age at onset of any allergic disease of 26.3 (22) years, defined as the earliest age at which any allergic disease (asthma, hay fever or eczema) was first reported (see **S1 Fig** for distribution).

**Fig 1:**
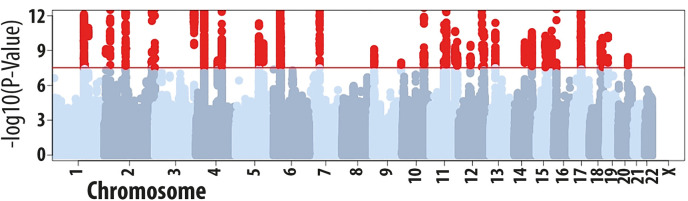
Summary of results from the GWAS of allergic disease age-of-onset in the UK Biobank study (*n* = 117,130). UK Biobank participants reported age-of-onset for asthma and, in a single separate question, for hay fever/eczema. In this analysis, we took the earliest age-of-onset reported across these two questionnaire items and tested this phenotype for association with SNP allelic dosage. We identified 4,160 variants associated with age-of-onset at a *P*<3x10^-8^ (red circles), including 50 with a statistically independent association.

We first performed a GWAS of a combined age-of-onset phenotype (*n* = 117,130 from the UK Biobank study. After adjusting the association results (**S2 Fig**) for the observed LD-score regression intercept [30] of 1.025, we identified 4,160 variants with a genome-wide significant association with age-of-onset (*P*<3x10^-8^, **Fig 1**). Of these, 50 variants in 40 loci (*i*.*e*. regions >1 Mb apart) remained associated at that threshold after accounting for the effects of adjacent SNPs in joint association analysis (<10 Mb; **Table 1** and **S3 Table**), indicating that they represent statistically independent associations with age-of-onset. Henceforth, we refer to these SNPs as sentinel variants for age-of-onset. Two additional variants had a *P*<3x10^-8^ in the joint but not in the original single-SNP analysis (**S4 Table**), both located in the major histocompatibility complex (MHC) locus. These represent secondary association signals at the MHC that were masked in the original GWAS by the association with other stronger nearby SNPs.

**Table 1:** Variants independently associated with allergic disease age-of-onset at a *P*<3x10^-8^.

Chr	Bp	Sentinel SNP	Gene context	Association with allergic disease age-of-onset (n = 117,130)					Novel association for allergic disease
				Effect allele	Freq#	Beta	SE	P-value	
1	152029548	rs115045402	S100A11-[]-TCHHL1	A	0.028	-0.202	0.014	1.7e-47	No
1	153051661	rs184587444	[SPRR2A]	T	0.020	-0.191	0.017	3.8e-28	Yes
1	155142927	rs4971089	[KRTCAP2]	G	0.488	-0.024	0.004	1.6e-08	Yes
1	172715702	rs78037977	FASLG-[]—TNFSF18	A	0.879	-0.044	0.006	1.1e-11	No
1	173141960	rs7521390	TNFSF18—[]-TNFSF4	C	0.296	-0.031	0.005	2.2e-11	No
2	8451701	rs13398375	[LINC00299]	T	0.716	-0.029	0.005	7.5e-10	No
2	28644670	rs7559046	FOSL2-[]-PLB1	C	0.537	-0.031	0.004	3.1e-13	No
2	228670437	rs10187276	SLC19A3-[]-CCL20	T	0.256	-0.034	0.005	4.6e-13	No
2	242698640	rs34290285	[D2HGDH]	G	0.757	-0.036	0.005	2.6e-13	No
3	188132110	rs6780858	[LPP]	G	0.524	-0.031	0.004	1.3e-13	No
4	38792340	rs6531663	TLR10-[]-TLR1	T	0.808	-0.067	0.005	2.6e-38	No
4	103515055	rs4648052	[NFKB1]	G	0.633	-0.025	0.004	7.0e-09	No
4	123141070	rs45613035	[KIAA1109]	C	0.104	-0.038	0.007	2.2e-08	No
4	123403008	rs45610037	IL2-[]—IL21	A	0.227	-0.042	0.005	1.5e-17	No
5	110164674	rs7728612	SLC25A46-[]—TSLP	T	0.167	-0.045	0.006	9.9e-16	No
5	110470137	rs6594499	WDR36-[]-CAMK4	C	0.530	-0.029	0.004	4.5e-12	No
5	132028858	rs4705962	[KIF3A]	T	0.234	-0.031	0.005	2.4e-10	No
6	31323012	rs2854001	[HLA-B]	A	0.237	-0.035	0.005	4.5e-13	No
6	32626015	rs6905282	HLA-DQA1-[]-HLA-DQB1	A	0.466	-0.042	0.004	1.1e-23	No
6	33033710	rs73739621	[HLA-DPA1]	C	0.096	-0.051	0.008	3.7e-11	No
7	50325815	rs2085423	C7orf72—[]-IKZF1	A	0.248	-0.035	0.005	4.1e-13	No
9	136155000	rs635634	ABO-[]-SURF6	T	0.189	-0.031	0.005	1.1e-08	No
10	104285594	rs12572775	[SUFU]	A	0.447	-0.033	0.004	7.6e-15	No
11	65559266	rs10791824	[OVOL1]	G	0.575	-0.035	0.004	1.8e-16	No
11	76299431	rs55646091	WNT11—[]-LRRC32	A	0.056	-0.089	0.009	4.7e-22	No
11	118746769	rs4938576	DDX6-[]-CXCR5	G	0.592	-0.028	0.004	2.1e-11	No
11	128161142	rs61907712	KIRREL3-AS3—[]—ETS1	C	0.813	-0.034	0.005	1.8e-10	No
12	56384804	rs705699	[RAB5B]	A	0.432	-0.025	0.004	3.6e-09	No
12	57493727	rs3024971	[STAT6]	T	0.900	-0.044	0.007	3.3e-10	No
12	111973358	rs597808	[ATXN2]	G	0.523	-0.033	0.004	1.9e-15	No
12	121202664	rs9431	[SPPL3]	A	0.497	-0.028	0.004	2.4e-11	No
13	43034968	rs1853573	AKAP11—[]—TNFSF11	G	0.465	-0.029	0.004	4.2e-12	No
14	68760527	rs7140939	[RAD51B]	A	0.403	-0.027	0.004	1.9e-10	No
14	103256961	rs56101042	[TRAF3]	A	0.820	-0.036	0.005	2.6e-11	No
15	61069988	rs11071559	[RORA]	C	0.877	-0.042	0.006	5.3e-11	No
15	67455630	rs56062135	[SMAD3]	T	0.247	-0.029	0.005	2.7e-09	No
15	90936225	rs2601191	[IQGAP1]	T	0.473	-0.028	0.004	1.9e-11	No
16	11229589	rs2041733	[CLEC16A]	T	0.447	-0.031	0.004	2.2e-13	No
17	38756969	rs7216890	CCR7-[]-SMARCE1	T	0.654	-0.033	0.004	7.2e-14	No
18	51780408	rs3017289	MBD2-[]-POLI	C	0.289	-0.029	0.005	2.4e-10	No
18	60009814	rs4574025	[TNFRSF11A]	T	0.540	-0.032	0.004	2.7e-14	No
18	61442619	rs12964116	[SERPINB7]	G	0.037	-0.108	0.011	6.2e-23	No
19	8785744	rs2918302	ADAMTS10—[]-ACTL9	A	0.156	-0.038	0.006	5.4e-11	No
20	45689783	rs4809619	[EYA2]	G	0.752	-0.029	0.005	3.7e-09	Yes
1	152179152	rs12123821	RPTN-[]-HRNR	T	0.051	-0.138	0.009	2.9e-48	No
1	152285861	rs61816761	[FLG]	A	0.026	-0.266	0.014	2.8e-82	No
2	102928617	rs72823628	[IL18R1]	G	0.874	-0.076	0.006	2.4e-33	No
9	6213468	rs7848215	RANBP6—[]-IL33	T	0.273	-0.029	0.005	7.5e-10	No
11	76295598	rs11236791	WNT11—[]-LRRC32	A	0.469	-0.046	0.004	3.8e-28	No
17	38067020	rs4795400	[GSDMB]	C	0.541	-0.050	0.004	6.0e-33	No

# Frequency of effect allele in the allergic disease cases studied.

Three of the 50 sentinel variants were in linkage disequilibrium (LD; *r*^2^>0.8) with variants previously reported to have a genome-wide significant association with asthma age-of-onset [29]: rs72823628 in *IL18R1*, rs7848215 near *IL33* and rs4795400 in *GSDMB*. Similarly, an additional three variants were in LD with SNPs that we reported recently [22] to be associated with the same combined age-of-onset phenotype: rs61816761 in *FLG*, rs12123821 near *HRNR* and rs11236791 near *LRRC32*. On the other hand, to our knowledge, the remaining 44 sentinel variants have not previously been implicated in the age-of-onset of any allergic disease at the genome-wide significance level.

Of the 12 specific variants previously reported to associate with allergic disease age-of-onset, 11 were tested in our current age-of-onset GWAS, of which nine had a highly significant and directionally concordant association (**S5 Table**). For two variants, there was no evidence for association with the combined age-of-onset phenotype: rs1861760 near *CYLD* (*P* = 0.41), reported by Sarnowski et al. [29], and rs9815663 near *CRBN* (*P* = 0.67), reported by Forno et al. [28]. The second variant reported by Forno et al. had a MAF<1% and so it was not tested in our current age-of-onset GWAS. We did however test this variant ad-hoc and found that it was not significantly associated with age-of-onset (*P* = 0.35, not shown).

### Potential impact of recall bias and phenotypic misclassification on SNP associations

All UK Biobank participants included in our analyses were adults (aged 38 to 70) at the time of data collection, and so recall bias might have affected the reported age-of-onset. Furthermore, proportionally, there were many individuals who reported late onset of allergic disease (*e*.*g*. 41% of asthmatics with onset ≥40 years old), which could have resulted from recall bias and/or phenotypic misclassification. We performed an additional set of analyses to determine if these potential confounders were likely to have had a major impact on the SNP associations described above. We addressed reliability of the age-of-onset information by comparing the self-reported age-of-onset between two surveys that were between 4–7 years apart. Age of onset was within 5 years accurate in 86% of cases. Subjects that reported less reliable information were likely to be older at enrollment. Older subjects were also less likely to report childhood onset asthma. When we analyzed the 50 sentinel variants in subjects who reported developing asthma as a child, and secondly, rhinitis as a child, we obtained highly consistent results, see **S4A Fig** and **S4B Fig**, respectively. We also replicated our findings in a prospective birth cohort ALSPAC, and show a high correlation of 0.67–0.825 of the effect size of our analysis with the results obtained in the ALSPAC study. Since the ALSPAC study prospectively assessed asthma, recall bias in this study is not a concern. Moreover, we correlated our findings for adult-onset asthma with two independent, published datasets of asthma GWAS performed by the GABRIEL consortium [26] and the TAGC consortium [31], and identified a substantial genetic correlation of r_g_ of 0.62 and 0.66, respectively. We further correlated our UKBB results of adult onset asthma with an analysis of adult onset asthma in the HUNT study, and again observed a significant genetic correlation r_g_ of 0.69. Further details of these analyses are provided in **S1 Data** (page 9) and **S3 Fig**–**S6 Fig**, and **S14 Table**.

### Association with age-of-onset in individuals suffering from a single allergic disease

By analyzing an age-of-onset phenotype that considered information from asthma, hay fever and eczema, the GWAS described above was expected to identify variants that affect age-of-onset broadly across the three diseases. To formally address this possibility, we tested each of the 50 sentinel variants identified above for association with the age-of-onset of asthma, hay fever and eczema, in three separate analyses. Specifically, we analysed age-of-onset in three non-overlapping groups of individuals (**S1 Fig**): those who reported suffering only from asthma (*n* = 22,029), only from hay fever (*n* = 14,474) or only from eczema (n = 3,969). Within each of these groups, we tested the association between the 50 sentinel variants and disease age-of-onset, using BOLT-LMM. In individuals suffering from asthma only, 19 sentinel variants were associated with variation in age-of-onset at *P*<3.3x10^-4^ (43 at *P*<0.05), which corrects for 50 SNPs tested in 3 groups, despite the smaller sample size of this analysis (**S6 Table**). For hay fever and eczema, there were respectively 8 and 5 SNPs associated with age-of-onset at that significance threshold (24 and 12 at *P*<0.05). Of note, the directional effect observed with the combined phenotype was the same as in the single disease analyses for most sentinel variants (100% for asthma, 94% for hay fever and 80% for eczema).

**Fig 2:**
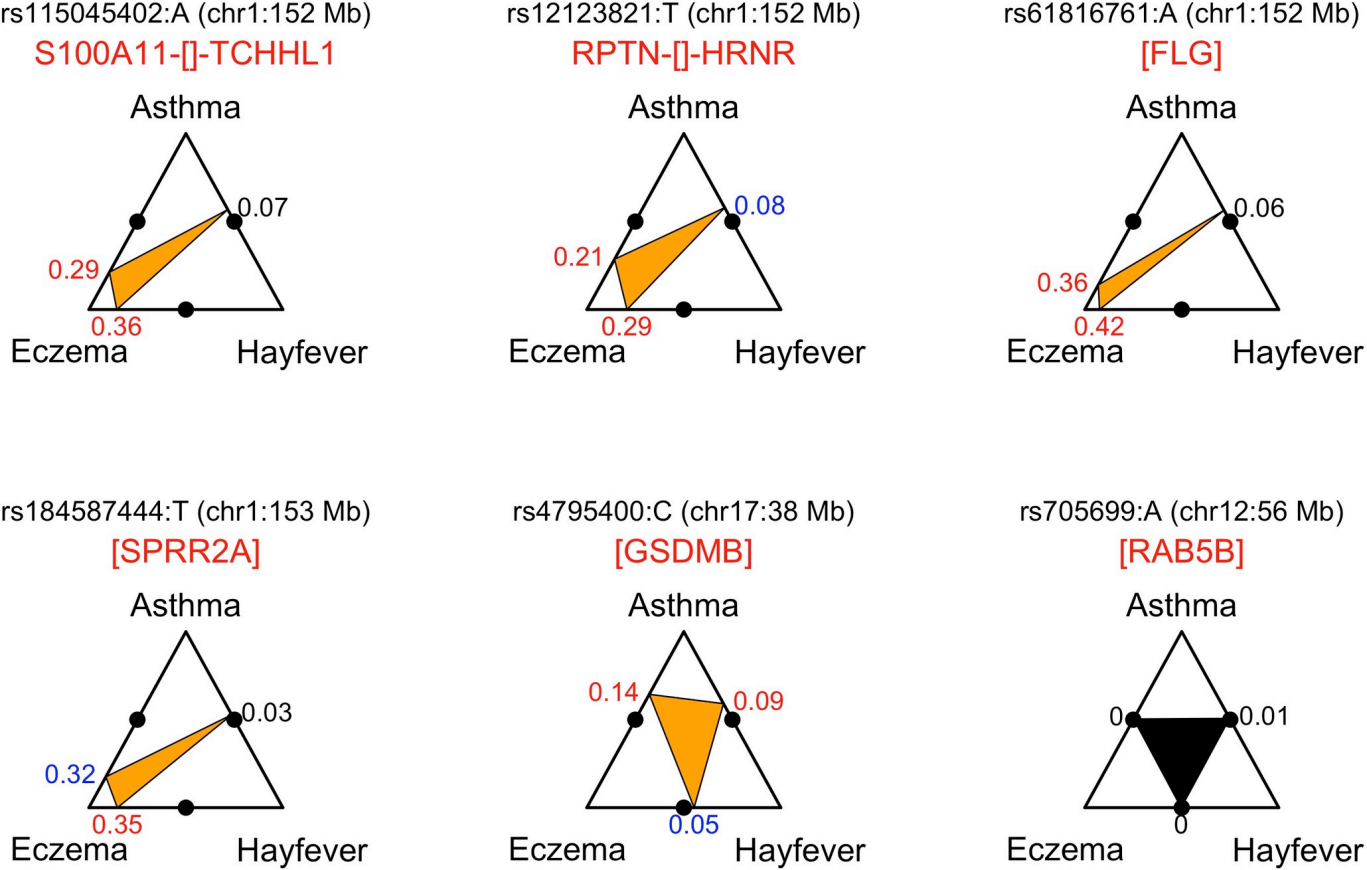
Variants with evidence for disease-specific effects on age-of-onset. Each of the 50 variants identified in the GWAS of age-of-onset were tested for association with age-of-onset in three non-overlapping groups of individuals: those suffering from asthma only (*n* = 22,029), hay fever only (*n* = 14,474) and eczema only (*n* = 3,969). We then compared the effects (*i*.*e*. betas) obtained in these three groups. For 5 of the 50 variants (shown with an orange inner triangle), the effect on age-of-onset was significantly different (*P*<0.05/(3 x 50) = 3.3x10^-4^) between at least two groups. For a given variant, the vertices of the inner triangle point to the position along the edges of the outer triangle that corresponds to difference in effect observed between pairs of single-disease cases. For example, the rs61816761[A] allele, which is located in the *FLG* gene (filaggrin), had an effect on age-of-onset that was larger (absolute of difference = 0.42) in individuals suffering only from eczema when compared to individuals suffering only from hay fever (*P* = 4.3x10^−8^), consistent with this SNP having a stronger effect on the age-of-onset of eczema than of hay fever. For comparison, a variant with no significant differences when comparing the effect on age-of-onset in all three pairwise single-disease association analyses is also shown (rs705699, in the *RAB5B* gene). In this case, the difference in effect was approximately equal to 0 in the three pairwise comparisons. The color of the difference in effect reflects the significance of the corresponding *z*-score (see Methods): red for *P*< 3.2x10^−4^ (correction for multiple testing), blue for *P*<0.05 and black for *P*>0.05.

Lastly, when we formally compared the effect of each sentinel variant on age-of-onset (*i*.*e*. the beta from the linear model) between pairs of diseases, we found that most variants (45 of 50, 90%) did not have significant disease-specific effects on age-of-onset (all pairwise comparisons with *P*>3.3x10^-4^; **S6 Table**). The exceptions were four variants located on chromosomes 1q21.3 (in/near *TCHHL1*, *HRNR*, *FLG* and *SPRR2A*) which had significantly stronger effects on age-of-onset of eczema, and one on 17q12 (in *GSDMB*) which had a stronger effect on the age-of-onset of asthma (**Fig 2**). Therefore, we conclude that most (45 of 50) sentinel variants identified in the GWAS of the combined age-of-onset phenotype have similar effects when considering the age-of-onset separately for asthma, hay fever and eczema.

### Association between age-of-onset sentinel variants and allergic disease risk

We then asked if the 50 sentinel variants were also likely to influence the risk of developing allergic disease, in addition to contributing to variation in age-of-onset amongst affected individuals. To this end, we investigated the association between each sentinel variant and a combined allergic disease phenotype, as reported in our recent GWAS [22]. After excluding the UK Biobank study from that GWAS, association results were based on data from 137,883 cases with asthma and/or hay fever and/or eczema, and 84,601 disease-free controls. Forty-eight of the 50 sentinel variants were tested in that GWAS, either directly or via a proxy (one variant), of which 38 (or 79%) were significantly associated with disease risk (*P*<0.001, which corrects for 48 tests; **Table 2**). This includes 19 variants for which the association with disease risk was genome-wide significant (*P*<3x10^-8^); that is, variants that represent previously known risk factors for allergic disease. Notably, for all 48 variants tested, the allele associated with a higher disease risk was associated with a lower age-of-onset. Therefore, we conclude that the sentinel variants identified influence both the likelihood of developing any allergic disease as well as the age at which symptoms first develop.

**Table 2:** Association between sentinel age-of-onset variants and allergic disease risk in an independent sample of 222,484 individuals studied by Ferreira et al. [22].

Chr	Bp	Sentinel SNP	Gene context	Association with allergic disease risk in Ferreira et al. 2017 (n = 222,484)*			
				Effect allele	Odds ratio	SE	P-value
1	152029548	rs115045402	S100A11-[]-TCHHL1	NA	NA	NA	NA
1	152179152	rs12123821	RPTN-[]-HRNR	T	1.091	0.019	2.40E-06
1	152285861	rs61816761	[FLG]	A	1.26	0.036	8.90E-11
1	153051661	rs184587444	[SPRR2A]	NA	NA	NA	NA
1	155142927	rs4971089	[KRTCAP2]	G	1.006	0.007	3.50E-01
1	172715702	rs78037977	FASLG-[]—TNFSF18	A	1.051	0.011	6.70E-06
1	173141960	rs7521390	TNFSF18—[]-TNFSF4	C	1.047	0.008	7.90E-10
2	8451701	rs13398375	[LINC00299]	T	1.063	0.008	1.20E-15
2	28644670	rs7559046*	FOSL2-[]-PLB1	C	1.034	0.007	3.50E-06
2	102928617	rs72823628	[IL18R1]	G	1.123	0.01	3.50E-32
2	228670437	rs10187276	SLC19A3-[]-CCL20	T	1.034	0.008	5.70E-05
2	242698640	rs34290285	[D2HGDH]	G	1.08	0.011	2.00E-13
3	188132110	rs6780858	[LPP]	G	1.036	0.007	5.00E-07
4	38792340	rs6531663	TLR10-[]-TLR1	T	1.088	0.008	3.30E-26
4	103515055	rs4648052	[NFKB1]	G	1.036	0.007	8.70E-07
4	123141070	rs45613035	[KIAA1109]	C	1.05	0.013	9.50E-05
4	123403008	rs45610037	IL2-[]—IL21	A	1.069	0.008	6.20E-16
5	110164674	rs7728612	SLC25A46-[]—TSLP	T	1.066	0.009	1.80E-12
5	110470137	rs6594499	WDR36-[]-CAMK4	C	1.073	0.007	1.70E-24
5	132028858	rs4705962	[KIF3A]	T	1.047	0.008	2.30E-08
6	31323012	rs2854001	[HLA-B]	A	1.061	0.009	5.30E-11
6	32626015	rs6905282	HLA-DQA1-[]-HLA-DQB1	A	1.063	0.007	3.70E-17
6	33033710	rs73739621	[HLA-DPA1]	C	1.06	0.013	1.10E-05
7	50325815	rs2085423	C7orf72—[]-IKZF1	A	1.017	0.008	3.60E-02
9	6213468	rs7848215	RANBP6—[]-IL33	T	1.07	0.008	3.80E-18
9	136155000	rs635634	ABO-[]-SURF6	T	1.039	0.009	9.60E-06
10	104285594	rs12572775	[SUFU]	A	1.015	0.007	2.60E-02
11	65559266	rs10791824	[OVOL1]	G	1.033	0.007	6.30E-06
11	76295598	rs11236791	WNT11—[]-LRRC32	A	1.088	0.007	3.70E-34
11	76299431	rs55646091	WNT11—[]-LRRC32	A	1.188	0.018	1.10E-22
11	118746769	rs4938576	DDX6-[]-CXCR5	G	1.037	0.007	2.30E-07
11	128161142	rs61907712	KIRREL3-AS3—[]—ETS1	C	1.041	0.009	5.60E-06
12	56384804	rs705699	[RAB5B]	A	1.039	0.007	6.80E-08
12	57493727	rs3024971	[STAT6]	T	1.083	0.012	4.00E-12
12	111973358	rs597808	[ATXN2]	G	1.029	0.007	3.50E-05
12	121202664	rs9431	[SPPL3]	A	1.028	0.007	4.50E-05
13	43034968	rs1853573	AKAP11—[]—TNFSF11	G	1.017	0.007	1.20E-02
14	68760527	rs7140939	[RAD51B]	A	1.033	0.008	1.60E-05
14	103256961	rs56101042	[TRAF3]	A	1.026	0.009	2.90E-03
15	61069988	rs11071559	[RORA]	C	1.055	0.01	5.30E-08
15	67455630	rs56062135	[SMAD3]	T	1.062	0.008	1.70E-13
15	90936225	rs2601191	[IQGAP1]	T	1.023	0.007	1.40E-03
16	11229589	rs2041733	[CLEC16A]	T	1.05	0.007	1.10E-12
17	38067020	rs4795400	[GSDMB]	C	1.066	0.007	5.70E-21
17	38756969	rs7216890	CCR7-[]-SMARCE1	T	1.026	0.007	2.00E-04
18	51780408	rs3017289	MBD2-[]-POLI	C	1.013	0.008	7.40E-02
18	60009814	rs4574025	[TNFRSF11A]	T	1.028	0.007	4.80E-05
18	61442619	rs12964116	[SERPINB7]	G	1.04	0.021	6.00E-02
19	8785744	rs2918302	ADAMTS10—[]-ACTL9	A	1.023	0.01	1.50E-02
20	45689783	rs4809619	[EYA2]	G	1.013	0.008	9.70E-02

# Association results from 12 of the 13 individual studies reported in the Ferreira et al. 2017 allergic disease GWAS were included in this analysis (all except UK Biobank). Results from the individual studies were adjusted for the respective study-specific LD-score intercept and then combined using a fixed-effects meta-analysis, as described previously [22]. The LD-score intercept of this 12-study meta-analysis was 1.018 (attenuation ratio of 0.0717)

* rs7559046 was not directly tested in the Ferreira et al. 2017 GWAS, and so we used a proxy instead (rs6547850, *r*^*2*^ = 0.93).

### Genetic correlation between age-of-onset and disease case-control status

For all 50 sentinel age-of-onset variants, the allele that was associated with a lower age-of-onset was associated with a higher risk of allergic disease. This observation suggested that these two traits–age-of-onset and case-control status of allergic disease–have a substantial negative genetic correlation; to our knowledge, this has not been previously estimated. To understand the extent to which the same genetic variants contribute to variation in these two traits, we applied LD-score regression [30] to the summary statistics of our age-of-onset and allergic disease [22] GWAS. Based on 1.1 million common SNPs, the genetic correlation between the two traits was estimated to be -0.625 (SE = 0.038, *P* = 4.5x10^-61^). This estimate was not expected to be biased by the sample overlap between the two GWAS [32], which we confirmed when we excluded samples from the UK Biobank study from the allergic disease [22] GWAS (*r*_*g*_ = -0.612, SE = 0.046, *P* = 5.0x10^-41^). These results indicate that a substantial fraction of genetic variants are likely to influence both the liability to, and the age-of-onset of, allergic disease. Furthermore, for most (but not necessarily all) shared variants, the directional effect is such that variants that are associated with higher disease risk are associated with lower age-of-onset.

**Fig 3:**
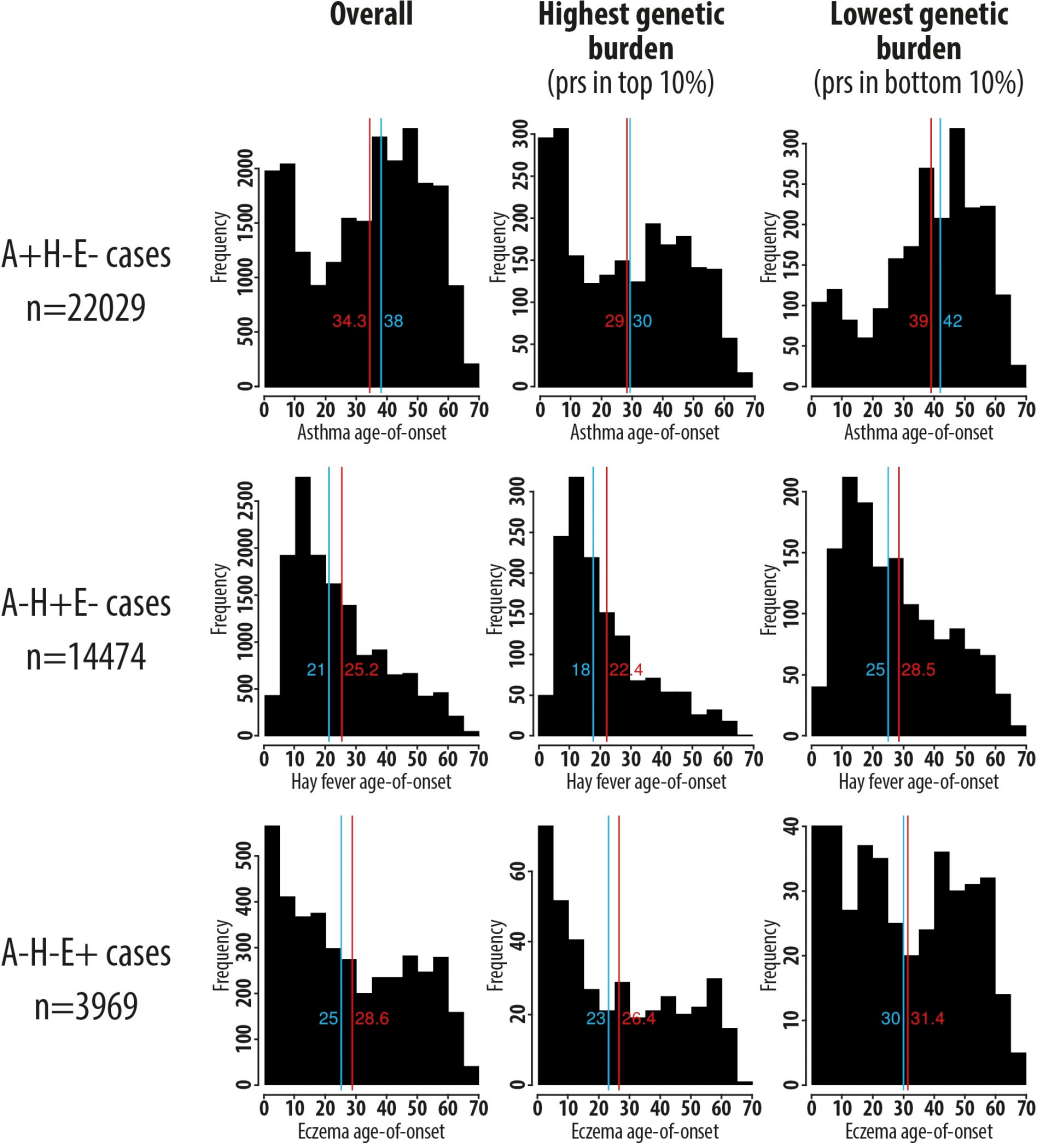
Distribution of allergic disease age-of-onset as a function of a polygenic risk score (PRS) for allergic disease in UK Biobank participants who reported suffering from a single disease (asthma only, hay fever only and eczema only). The PRS of each individual was calculated based on 136 SNPs that were associated with allergic disease risk in our recent GWAS [22]. The mean and median of each distribution are shown in red and blue, respectively.

More broadly, these results strongly suggest that a key risk factor that distinguishes individuals with early disease onset from those with late disease onset is the overall genetic burden inherited at allergy-associated SNPs. To illustrate this effect, we compared the distribution of age-of-onset between individuals with the highest (top 10%) and the lowest (bottom 10%) polygenic risk score (PRS) for allergic disease, constructed for each individual from the UK Biobank study based on information from 136 allergy risk variants that we reported recently [22]. This analysis was performed separately for asthma, hay fever and eczema, using the same single-disease case groups described above. For asthma, individuals with the lowest genetic burden of allergic disease (*n* = 2,202) had a median age-of-onset of 39 years, with only 14% having an age-of-onset before the age of 16; the distribution of age-of-onset was broadly consistent with a pattern of late disease onset (**Fig 3**). In contrast, in the group with the highest genetic burden (*n* = 2,203), the median age-of-onset decreased to 29 years, with 35% of individuals reporting that asthma was diagnosed before the age of 16. In this group, there was a clear shift in the distribution of age-of-onset towards a pattern of early disease onset. Similar results were observed for hay fever and eczema (**Fig 3**). Collectively, our results indicate that genetic risk factors for allergic disease are enriched in cases with early disease onset when compared to those with late disease onset.

### Multivariate GWAS of allergic disease case-control status and age-of-onset

**Fig 4:**
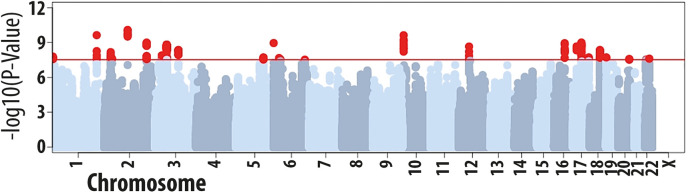
Summary of results from the multivariate analysis of allergic disease age-of-onset and allergic disease case-control status. The GWAS of allergic disease age-of-onset was performed in the UK Biobank study (*n* = 117,130) as described in the main text. The GWAS of allergic disease case-control status included 360,838 individuals, has reported recently [22]. Single-SNP results from each GWAS were adjusted for the top independent associations (*P*<3x10^-8^) identified and then multivariate analysis was performed using metaUSAT [33]. We identified 281 variants with a multivariate *P*<3x10^-8^ (red circles), including 26 that were in low LD (*r*^*2*^<0.05) with each other and so that are likely to represent statistically independent associations.

The high genetic correlation observed between case-control status and age-of-onset of allergic disease suggests that a large number of variants contribute to the heritability of both traits. We therefore hypothesized that multivariate association analysis would identify variants jointly associated with both traits that were missed in the single-trait analyses. To this end, we first adjusted the single-SNP results obtained in the age-of-onset and case-control [22] GWAS for the effects of the sentinel variants identified in the respective study. In the two resulting adjusted GWAS, there were no variants with an association significant at a *P*<3x10^-8^, as expected (**S6 Fig and S7 Fig)**. There was, however, an excess of significant associations when compared to the number expected by chance given the number of SNPs tested (**S8 Fig and S9 Fig).** Many of these associations are likely to represent true positive findings that do not reach the stringent genome-wide significance threshold in each of those two univariate analyses. To help identify these, we then performed multivariate analysis of age-of-onset and case-control status, using metaUSAT [33], which is applicable to association summary statistics. Using this approach, we identified 281 variants with a multivariate *P*<3x10^-8^ (**Fig 4** and **S11 Fig**), including 26 that were in low LD with each other (*r*^2^<0.05) and so that are likely to represent statistically independent associations (**Table 3**). However, the QQ Plots may indicate some inflation of the P values, so therefore, these data need to be interpreted with caution. The genomic inflation factor could not be calculated because metaUSAT does not have a closed form null distribution. Nonetheless, inflation of significant associations can be assessed by comparing the observed and expected number of associations significant at a given significance threshold. We observed 38%, 17%, 10%, 5.9% and 1.9% of SNPs tested with a multivariate P-value <0.5, <0.2, <0.1, <0.05 and <0.01, respectively, when the expectations under the null hypothesis of no association were 50%, 20%, 5% and 1%. For most variants, the association in each of the two univariate analyses was one to four orders of magnitude below genome-wide significance, which was exceeded in the multivariate analysis. For all variants, the allele associated with higher disease risk was associated with lower age-of-onset. Results obtained with the recently described MTAG multivariate approach [34] supported the associations identified with metaUSAT (**S7 Table**). We conclude that these 26 variants represent risk factors for both the presence and early onset of allergic disease, which were only detectable when we combined information from the age-of-onset and case-control GWAS.

**Table 3:** Variants jointly associated with allergic disease age-of-onset and allergic disease risk (multivariate *P*<3x10^-8^).

Chr	Bp	Sentinel SNP	Gene Context	metaUSAT P-value	Effect allele and frequency		Association with allergic disease age-of-onset (n = 117,130)			Association with allergic disease risk in Ferreira et al. 2017 (n = 360,838)			Novel
							Beta	SE	P	Beta	SE	P	
1	2510755	rs10910095	TNFRSF14-[]-FAM213B	1.62e-08	G	0.868	-0.017	0.006	6.0e-03	0.041	0.007	4.3e-08	No
1	212864992	rs12068304	[BATF3]	2.32e-10	G	0.166	-0.026	0.006	3.9e-06	0.035	0.007	5.8e-07	No
2	30846848	rs7565907	[LCLAT1]	6.66e-09	T	0.609	-0.017	0.004	5.5e-05	0.025	0.005	1.4e-06	Yes
2	37137123	rs112844988	[STRN]	2.48e-08	G	0.368	-0.020	0.004	2.2e-06	0.02	0.005	1.4e-04	Yes
2	112268732	rs143326447	BCL2L11—[]—ANAPC1	8.22e-11	C	0.123	-0.030	0.006	3.2e-06	0.043	0.008	2.3e-07	No
2	203487023	rs72926957	BMPR2-[]-FAM117B	1.01e-09	G	0.706	-0.023	0.005	4.7e-07	0.023	0.006	2.3e-05	Yes
3	33047662	rs35570272	[GLB1]	1.28e-08	T	0.404	-0.023	0.004	8.2e-08	0.016	0.005	2.3e-03	No
3	56605990	rs6778373	[CCDC66]	1.51e-09	A	0.535	-0.017	0.004	7.5e-05	0.026	0.005	2.2e-07	Yes
3	112643560	rs9870568	[CD200R1]	4.35e-09	C	0.474	-0.016	0.004	1.7e-04	0.026	0.005	3.1e-07	Yes
5	131952222	rs6596086	[RAD50]	1.9e-08	C	0.200	-0.018	0.005	7.5e-04	0.032	0.006	3.4e-07	No
6	209159	rs11242709	[]-DUSP22	1.11e-09	T	0.210	-0.029	0.005	4.3e-08	0.024	0.007	3.0e-04	Yes
6	26186200	rs9379832	HIST1H2BE-[]-HIST1H4D	2.15e-08	G	0.26	-0.019	0.005	6.5e-05	0.027	0.006	4.2e-06	Yes
6	32624874	rs28483633	HLA-DQA1-[]-HLA-DQB1	2.77e-08	C	0.838	-0.035	0.007	1.5e-07	0.056	0.019	2.8e-03	No
6	149909491	rs9322188	[GINM1]	2.99e-08	T	0.313	-0.015	0.005	7.3e-04	0.028	0.006	5.7e-07	Yes
10	6625378	rs2255088	[PRKCQ-AS1]	2.35e-10	C	0.335	-0.021	0.004	1.5e-06	0.027	0.006	1.5e-06	No
12	50336638	rs416959	LOC283332-[]-AQP2	2.21e-09	T	0.824	-0.026	0.006	3.4e-06	0.030	0.007	7.2e-06	Yes
16	50745926	rs2066844	[NOD2]	1.13e-09	T	0.049	-0.043	0.010	8.2e-06	0.059	0.012	1.5e-06	No
16	50885211	rs8056255	CYLD-[]—SALL1	3.26e-09	A	0.033	-0.047	0.012	5.5e-05	0.071	0.014	6.8e-07	Yes
17	17624349	rs77904527	[RAI1]	2.17e-09	C	0.147	-0.031	0.006	2.5e-07	0.028	0.007	1.0e-04	Yes
17	40741013	rs12951632	[FAM134C]	1e-09	T	0.719	-0.026	0.005	3.4e-08	0.020	0.006	3.4e-04	No
17	76244926	rs72901762	TMEM235-[]-LOC100996291	1.89e-08	A	0.711	-0.019	0.005	3.5e-05	0.026	0.006	6.7e-06	Yes
18	48558415	rs35014537	[SMAD4]	4.34e-09	G	0.384	-0.018	0.004	3.1e-05	0.025	0.005	1.6e-06	No
19	1170445	rs4807630	[SBNO2]	1.89e-08	T	0.312	-0.020	0.005	1.5e-05	0.026	0.006	1.6e-05	No
20	52258875	rs4811448	ZNF217-[]—SUMO1P1	2.6e-08	C	0.293	-0.017	0.005	2.9e-04	0.028	0.006	1.2e-06	Yes
22	21939675	rs5754217	[UBE2L3]	2.93e-08	T	0.196	-0.015	0.005	3.8e-03	0.034	0.006	1.2e-07	Yes
22	37319947	rs4437064	[CSF2RB]	2.44e-08	G	0.529	-0.009	0.004	3.2e-02	0.028	0.005	3.0e-08	Yes

### Sentinel variants not previously implicated in the aetiology of allergic disease

We then determined which of the sentinel variants identified in the age-of-onset and multivariate GWAS described above represented novel associations for allergic disease in general, that is, when considering all previously reported associations with *P*<5x10^-8^ for asthma, hay fever, eczema, food allergy and/or atopy. Of the 50 sentinel variants identified in our age-of-onset GWAS, 47 were in LD (*r*^2^>0.05) with variants previously reported to associate with allergic disease (**S8 Table**). The remaining 3 represent novel associations for allergic disease: rs184587444 in *SPRR2A*, rs4971089 in *KRTCAP2*, and rs4809619 in *EYA2* (**Table 1**). On the other hand, most 15 of the 26 sentinel variants identified in the multivariate GWAS represent novel associations for allergic disease (**Table 1** and **S8 Table**), including for example rs7565907 in *LCLAT1*and rs11242709 near *DUSP22*. Thus, overall, by considering age-of-onset information, we identified 18 (3+15) novel genetic associations for allergic disease.

### Likely target genes of sentinel variants identified in the age-of-onset and multivariate GWAS

To help understand how the 76 sentinel variants might influence allergic disease pathophysiology, we identified genes for which variation in gene expression and/or protein sequence was associated/determined by SNPs in LD with the sentinel variants.

We first extracted association summary statistics from 101 published datasets of eQTL identified in five different broad tissue types relevant for allergic disease (**S1 Table**). For each gene and for a given eQTL dataset, we then (i) identified *cis* eQTL in low LD (*r*^2^<0.05) with each other, which we refer to as “sentinel eQTL”; and (ii) determined if any of the 76 sentinel variants were in high LD (*r*^2^>0.8) with a sentinel eQTL. Using this approach, we found sentinel eQTL in LD with 26 of the 50 (52%) sentinel variants identified in the age-of-onset GWAS (**S9 Table**), and with 15 of the 26 (58%) sentinel variants identified in the multivariate GWAS (**S10 Table**). The sentinel eQTL implicated respectively 47 and 28 genes (one in common: *HLA-DQB1*) as likely targets of the sentinel variants identified in these two GWAS (**Table 4**).

**Table 4:** Genes with a sentinel eQTL (in italic) or non-synonymous SNP (in bold) in LD (*r*^*2*^>0.8) with sentinel variants identified in the age-of-onset or multivariate GWAS.

Chr	Bp	Sentinel SNP	Gene context	LD between sentinel eQTL/non-synonymous SNP and sentinel GWAS SNP	
				*r*^*2*^ > 0.95	0.80 < *r*^*2*^ ≤ 0.95
*Sentinel variants identified in the GWAS of age-of-onset*					
1	152285861	rs61816761	[FLG]	**FLG**	*-*
1	155142927	rs4971089	[KRTCAP2]	*-*	*ADAM15*, **EFNA1**
1	173141960	rs7521390	TNFSF18—[]-TNFSF4	*TNFSF4*	*-*
2	28644670	rs7559046	FOSL2-[]-PLB1	*-*	*FOSL2*,*RP11-373D23*.*3*
2	102928617	rs72823628	[IL18R1]	*MFSD9*	*IL18RAP*,*IL1RL1*
2	228670437	rs10187276	SLC19A3-[]-CCL20	*CCL20*	*-*
3	188132110	rs6780858	[LPP]	*-*	*BCL6*
4	38792340	rs6531663	TLR10-[]-TLR1	***TLR1***	*-*
4	103515055	rs4648052	[NFKB1]	*-*	*NFKB1*
4	123403008	rs45610037	IL2-[]—IL21	*KIAA1109*	*-*
5	110470137	rs6594499	WDR36-[]-CAMK4	*-*	*CAMK4*,*CTC-551A13*.*2*,*TSLP*,*WDR36*
5	132028858	rs4705962	[KIF3A]	*-*	*KIF3A*
6	31323012	rs2854001	[HLA-B]	*-*	*HLA-C*
6	33033710	rs73739621	[HLA-DPA1]	*-*	*HLA-DPA1*,*HLA-DPB1*,*HLA-DQB1*,*TAPBP*
10	104285594	rs12572775	[SUFU]	*-*	*ACTR1A*,*C10orf32*,*SUFU*,*TMEM180*,*TRIM8*
11	65559266	rs10791824	[OVOL1]	*EFEMP2*,*OVOL1*,*SNX32*	*-*
12	56384804	rs705699	[RAB5B]	*RPS26*,*SUOX*	*-*
12	57493727	rs3024971	[STAT6]	*NAB2*,*STAT6*	*-*
12	111884608	rs597808	[ATXN2]	**SH2B3**	*-*
12	121202664	rs9431	[SPPL3]	*SPPL3*	*OASL*
13	43034968	rs1853573	AKAP11—[]—TNFSF11	*TNFSF11*	*-*
15	61069988	rs11071559	[RORA]	*-*	*RP11-554D20*.*1*
15	67455630	rs56062135	[SMAD3]	*AAGAB*	*-*
15	90936225	rs2601191	[IQGAP1]	*-*	*IQGAP1*
17	38067020	rs4795400	[GSDMB]	**GSDMB**	*GSDMB*,*IKZF3*,*ORMDL3*,*ZPBP2*
17	38756969	rs7216890	CCR7-[]-SMARCE1	*SMARCE1*	*-*
18	60009814	rs4574025	[TNFRSF11A]	*PIGN*	*-*
18	61442619	rs12964116	[SERPINB7]	*SERPINB7*	*-*
*Sentinel variants identified in the multivariate GWAS of age-of-onset and case-control status*					
1	2510755	rs10910095	TNFRSF14-[]-FAM213B	**TNFRSF14**	*-*
2	30846848	rs7565907	[LCLAT1]	*-*	*LCLAT1*
2	37137123	rs112844988	[STRN]	*STRN*	*-*
2	203487023	rs72926957	BMPR2-[]-FAM117B	*BMPR2*,*FAM117B*	*-*
3	56605990	rs6778373	[CCDC66]	***CCDC66***,***FAM208A***	*-*
3	112643560	rs9870568	[CD200R1]	***CD200R1***	*-*
5	131952222	rs6596086	[RAD50]	*SLC22A5*	*-*
6	26186200	rs9379832	HIST1H2BE-[]-HIST1H4D	*-*	**HIST1H2BE**
6	32624874	rs28483633	HLA-DQA1-[]-HLA-DQB1	*HLA-DQA1*,*HLA-DQB1*,*HLA-DQB2*	**HLA-DQA1**
6	149909491	rs9322188	[GINM1]	*PCMT1*	*GINM1*,*LATS1*,*NUP43*
10	6625378	rs2255088	[PRKCQ-AS1]	*PRKCQ*	*-*
16	50745926	rs2066844	[NOD2]	**NOD2**	*-*
16	50885211	rs8056255	CYLD-[]—SALL1	*-*	*NOD2*
17	40741013	rs12951632	[FAM134C]	*FAM134C*,***MLX***,*PSMC3IP*	*BECN1*
17	76244926	rs72901762	TMEM235-[]-LOC100996291	*RP11-219G17*.*4*	*THA1P*
18	48558415	rs35014537	[SMAD4]	*RP11-729L2*.*2*,*SMAD4*	*-*
19	1170445	rs4807630	[SBNO2]	*-*	*ABCA7*
22	21939675	rs5754217	[UBE2L3]	*CCDC116*,*UBE2L3*	**YDJC**

Second, we found 21 non-synonymous SNPs in 14 genes that were in high LD (*r*^2^>0.8) with sentinel variants identified in the age-of-onset or multivariate GWAS (**S11 Table**). This list included, for example, four non-synonymous SNPs in the *CD200R1* gene that were in complete LD (*r*^2^ = 1) with the sentinel variant identified in the multivariate GWAS. Of the 14 genes, seven were novel target predictions, that is, they were not identified in the eQTL analysis described above: *FLG*, *EFNA1*, *SH2B3*, *TNFRSF14*, *HIST1H2BE*, *MLX* and *YDJC*. Overall, when considering information from eQTL and non-synonymous SNPs, we identified 81 (47+27+7) likely target genes of the 76 sentinel variants identified in this study.

### Association between the 76 sentinel variants and the risk of food allergy

Finally, we tested if the sentinel variants identified above were associated with food allergy case-control status, in children and adults separately. Although the discovery analysis that identified the sentinel variants for age of onset of allergy did not include food allergy, we hypothesized that these sentinel variants may also relate to food allergy. First, we extracted association results from GWAS that we published recently [15], comprising 497 children with food allergy diagnosed by oral food challenge in the GOFA study and 2,387 controls. This study comprised a highly selected group of children with early onset food allergy (mean age at diagnosis was 2.1 years). In that GWAS, nine of the 76 sentinel variants were significantly associated with food allergy after correcting for multiple testing (*P*<0.05/76 = 0.00065; **S13 Table**), namely those in/near *FLG* (four variants), *KIF3A*, *LRRC32*, *RAD50*, *CYLD*, and *SERPINB7*. Overall, there was a very close agreement in SNP associations between the age-of-onset and food allergy analyses (**S12 Fig**); for example, for 66 of 76 variants the allele associated with a lower age-of-onset onset was associated with a higher disease risk (binomial test *P* = 2x10^-12^).

To assess the association between the 76 sentinel variants and food allergy risk in adults, we extracted association results from a GWAS of self-reported food allergy conducted in the adult GERA cohort [16], which included 5,108 subjects with self-reported food allergy, of whom 1,104 were admitted to hospital because of food allergy and 23,945 controls who did not report to have food allergy. In this GWAS, we compared the 1,104 subjects admitted to hospital because of food allergy to the 23.945 controls. No single variant was significantly associated with food allergy after correcting for multiple testing (**S13 Table**). Across the 72 variants tested in both the child and adult food allergy GWAS, only 43 (60%) had a directionally consistent association, reflecting very little agreement between results from the two analyses. Overall, our results show that many variants associated with allergic disease age-of-onset also represent genetic risk factors for food allergy in young children but not (or less so) in adults. Moreover, the self-report of food allergy in the adult population is more subject to misclassification and may also have contributed to this latter observation.

## Discussion

In this study, we identified (i) 50 variants associated with allergic disease age-of-onset; (ii) a significant negative genetic correlation between allergic disease age-of-onset and case-control status; (iii) 26 additional variants jointly associated with allergic disease age-of-onset and case-control status; (iv) 81 genes that are likely targets of sentinel variants identified in the age-of-onset or multivariate GWAS; and (v) nine variants (out of the 76) that are also associated with the risk of food allergy in young children.

Amongst the 50 associations for allergic disease age-of-onset, six were reported in previous studies of age-of-onset [28,29], but the remaining 44 were novel associations for this phenotype. Conversely, of the 12 variants reported in previous studies, nine were associated in our GWAS, but three were not (in/near *CYLD/NOD2* [29], *CRBN* [28] and *ETS1* [28]). Possible explanations for the lack of association with these three variants in our GWAS is that their effect on age-of-onset is population specific or specific to asthma, the disease considered in the original studies. However, the three variants were also not significantly associated with age-of-onset when we restricted our analysis to cases who suffered only from asthma (*P* = 0.17, *P* = 0.64 and *P* = 0.26, respectively), which suggests that disease-specific effects are unlikely to explain the discordance.

When we compared the effect of the 50 variants on the age-of-onset of each of the three individual diseases, we found significant differences only for five variants. Four of these had a stronger effect on the age-of-onset of eczema: those in/near *HRNR* (rs1213821), *FLG* (rs61816761), *TCHHL1* (rs115045402), *SPRR2A* (rs184587444), all within a 1 Mb locus on chromosome 1q21. The former two represent known risk factors for allergic disease, with a stronger effect on eczema [22], consistent with our results. On the other hand, the latter two variants, which are relatively uncommon (MAF of 2.8% and 2.0%), have not previously been established as risk factors for allergic disease, although our results for age-of-onset suggest that this is very likely to be the case. We did not find any eQTL in LD with either variant; on the other hand, both variants are in low to moderate LD with rs558269137 (*r*^2^ = 0.46 and 0.24, respectively), which encodes the *FLG* 2282del4 mutation that is associated with eczema and ichthyosis vulgaris [35]. It is therefore possible that at one (or both) of these variants are tagging that mutation, which was not tested in our study. The fifth variant, rs4795400 in *GSDMB*, showed a stronger effect on the age-of-onset of asthma. This variant is in high LD (*r*^2^>0.8) with variants reported to associate with earlier age-of-onset for asthma (rs9901146) [29] and which are stronger risk factors for asthma when compared to hay fever and eczema (rs921650) [22], consistent with our results. For the remaining 45 variants identified in our GWAS, our results suggest that their effect on age-of-onset is comparable between the three individual diseases.

We also investigated if the 50 variants that determined variation in age-of-onset amongst allergic disease cases also contributed to differences in case-control status amongst an independent sample of 222,484 individuals not part of the UK Biobank that we studied recently [22]. Remarkably, 39 of 48 variants with available results had a significant association with case-control status after accounting for multiple testing. Furthermore, for all 39 variants (and also for the other nine tested), the disease-predisposing allele was associated with a lower age-of-onset. These results suggested that case-control status and age-of-onset have a strong negative genetic correlation, which we confirmed (*r*_g_ = -0.63) using genome-wide SNP data. We highlight two implications that arise from this observation.

First, this observation confirms that many genetic variants, including those identified in our age-of-onset GWAS, determine both the lifetime risk of developing an allergic disease as well as the age at which symptoms first develop. As such, combining information from these two phenotypes can help identify variants that influence disease liability, as suggested previously [29]. Motivated by this prediction, we performed multivariate analysis of results from our GWAS of age-of-onset and our recently published GWAS of allergic disease case-control status, which also considered information from asthma, hay fever and eczema. Importantly, we used a multivariate approach (metaUSAT [33]) that was expected to increase power to detect an association with a variant that influences both traits, when compared to other methods that are also applicable to GWAS summary statistics (*e*.*g*. metaCCA [36]). In this analysis, we identified 26 variants that were missed by the individual GWAS, highlighting the substantial gain in power that can be obtained by combining information from age-of-onset and case-control status. Of these 26 variants, only six were in LD (*r*^2^>0.05) with variants previously reported in GWAS of allergic disease. Therefore, most represent new associations for both age-of-onset and disease risk. Since we were not able to formally replicate these findings in an independent study, we emphasize the importance of future studies to replicate our results. We also suggest that this approach could be extended to include other phenotypes that can be shown to have a significant genetic correlation with disease risk; for example, these could be disease severity or markers of allergic sensitization.

Second, the large negative genetic correlation between case-control status and age-of-onset indicates that for most variants associated with both traits, the allele that is more common in allergic disease cases (when compared to controls) is also more common in cases with early onset disease (when compared to those with late onset disease). That is, individuals who inherit a larger overall burden of allergy-predisposing alleles are more likely to have early onset disease when compared to those who inherit a lower genetic burden, consistent with previous findings [37]. This shows that allergic disease risk alleles are more common in early onset disease, which might imply that allergic disease with late onset is less heritable (i.e. more ‘environmental’) than allergic disease with early onset. For example, it is conceivable that in late onset disease, environmental (more than genetic) risk factors dysregulate the expression of genes that play a key role in disease pathophysiology through epigenetic mechanisms, as we suggested recently [22]. But that may not necessarily be the case. Instead, it is possible that individuals develop late onset disease because they inherit risk alleles that influence asthma, hay fever and/or eczema pathophysiology through mechanisms that are not shared with early onset disease. Studies that address these possibilities are warranted. It is also important to highlight that we cannot rule out the possibility that recall bias might have contributed to the negative genetic correlation observed between age-of-onset and case-control status. This might have occurred if recall bias was less common amongst subjects who reported a younger age of onset.

We used information from eQTL studies and non-synonymous SNPs to identify 81 genes that are likely targets of 48 of the 76 (63%) variants identified in either the age-of-onset or multivariate GWAS performed. In the **S1 Data** (page 10–15), we discuss in greater detail 10 genes that are plausible targets of novel allergic disease variants identified in our study and that have a known function that is directly relevant to disease pathophysiology. In brief, the 10 genes are: *ADAM15*, a metalloproteinase which cleaves the toll like receptor adaptor molecule TRIF [38] and the low affinity IgE receptor [39]; *FOSL2*, a regulator of cell proliferation involved in B cell, Th17 cell and epidermal differentiation and function [40–42]; *TRIM8*, a ligase involved in post-translational modifications of proteins, including ubiquitination of TAK1 [43] and TRIF [44]; *BMPR2*, a receptor for the TGF-beta superfamily [45] that inhibits Smad-mediated signaling [46]; *CD200R1*, a surface glycoprotein that interacts with CD200 [47], which is known to suppress the activation of various immune cells, including macrophages [48], mast cells [49], monocytes [50] and dendritic cells [51]; *PRKCQ*, a protein kinase involved in the development and function of Th17 cells [52], Th2 cells [53], Tregs [54] and type 2 innate lymphoid cells [55]; *NOD2*, an intracellular pattern recognition receptor that upon activation by bacterial peptidoglycans [56] and viruses [57] promotes host defense through the production of inflammatory mediators [58–60]; *SMAD4*, a central regulator of TGF-beta signaling [61], involved in Th2 cytokine production [62], Treg [63] and Th17 differentiation [64], the expression of selectin ligands [65] and of the pro-allergic cytokine IL-9 [66]; *ABCA7*, a transporter protein that moves lipids across membranes [67], enhances phagocytosis of apoptotic cells by macrophages [68], promotes NKT cell development and function [69], and was suggested to play a role in keratinocyte differentiation [70]; and *UBE2L3*, an essential component of the post-translational protein ubiquitination pathway, which plays a major role in the regulation of inflammatory responses [71–75].

The combined age-of-onset phenotype analysed did not take into account information from food allergy, as this was not available in the UK Biobank study. To partly address this limitation, we tested if the 76 sentinel variants identified in the age-of-onset or multivariate GWAS were also associated with food allergy, both in children and adults. After correcting for multiple testing, nine variants were significantly associated with food allergy confirmed by oral challenge in young children of the GOFA study [15], including one variant located in a locus not previously reported in food allergy GWAS: rs8056255 near *CYLD*. As such, this variant represents a putative novel risk factor for food allergy, which should be studied in greater detail in future studies. On the other hand, there was no evidence that the sentinel variants for age-of-onset identified in our study were associated with food allergies (based on hospital admissions) in adults of the GERA cohort. The lack of agreement between the food allergy results obtained in the GOFA and GERA studies raises the possibility that genetic risk factors for food allergy in children and adults might be largely distinct, which warrants further investigation.

Another potential limitation of our study is that age-of-onset reported by UK Biobank participants may have been affected by recall bias. For example, individuals with current disease symptoms at the time of data collection might have recalled early onset of disease more reliably than those who no longer suffered from allergies. We addressed this potential limitation by testing the association between the 50 sentinel variants identified in the age-of-onset GWAS in a subset of UK Biobank individuals who reported developing asthma as a child, specifically up to age 19. We found that the association between the 50 sentinel variants and age-of-onset in this smaller but more homogenous group of allergic disease cases was consistent with results obtained in the overall sample. Furthermore, we also found consistent associations when considering asthma onset recorded in children from the independent and prospective Avon Longitudinal Study of Parents and Children (ALSPAC) birth cohort. Similar results were observed for the 26 sentinel variants identified in the multivariate GWAS (**S14 Table**). Therefore, the 76 sentinel variants reported in our study show a consistent pattern of association with allergic disease age-of-onset in two analyses for which recall bias was not a major concern. Similarly, we found that phenotypic misclassification amongst individuals who reported late onset of allergic disease, if present, was unlikely to have significantly affected our main findings. In addition, the collection of information in adulthood is likely to have caused overrepresentation of SNPs involved in persistent disease and underrepresentation of association related to disease that remitted earlier in life (transient disease). Finally, we showed that in a subset of cases that provided data on two different occasions 4–7 years apart, age of onset of asthma was within 5 years in 86% of cases. However, we were not able to investigate this reliability for eczema and hayfever separately, since only a combined question was available. Furthermore, we acknowledge that only 3% of asthmatics in UKBB provided data on two different occasions. Thus, recall bias may have reduced our power, but not have resulted in spurious results.

In conclusion, we show that novel risk loci for allergic disease can be identified by extending the analytical approach that we reported recently [22] to the analysis of age-of-onset of asthma, hay fever and eczema. GWAS of other complex diseases might also benefit from considering age-of-onset information. We found 76 specific genetic associations with allergic disease, of which 28 had not previously been reported. We implicate 81 genes as likely targets of the associated variants and provide further evidence that individuals with early disease onset have a greater burden of genetic risk factors for allergic disease than individuals with late disease onset.

## Methods

### Definition of the combined age-of-onset phenotype and allergic disease status

We created a single age-of-onset phenotype for individuals from the UK Biobank study [76] that considered information from asthma, hay fever and eczema. Age-of-onset for food allergy was not available in the UK Biobank study and so was not considered in our analysis.

Specifically, we extracted information from two data fields included in the touchscreen questionnaire. Field 3786, which asked “What was your age when the asthma was first diagnosed?”, and field 3761, which asked “What was your age when the hayfever, rhinitis or eczema was first diagnosed?”. After excluding individuals who were not genotyped (absent from the array data), non-missing information was available for 50,109 and 98,161 individuals, respectively for fields 3786 and 3761. The combined age-of-onset phenotype corresponded to the earliest age reported across these two fields, which was obtained for 127,382 individuals. Of these, we restricted our analysis to individuals who were determined to suffer from at least one allergic disease (asthma and/or hay fever and/or eczema) based on the criteria described in detail previously [22]. Briefly, “asthma cases” were those with (i) both a report of “Asthma” in field 6152 (self-reported medical conditions, specifically the question “Has a doctor ever told you that you have had any of the following conditions?”) and a code for asthma in field 20002 (verbal interview), or alternatively, an ICD10 code for asthma in fields 41202 (Diagnoses–main ICD10) or 41204 (Diagnoses–secondary ICD10); and (ii) no report of COPD in fields 6152 or 20002, nor of other respiratory diseases in field 20002. “Hay fever/eczema cases” were those who answered “Hay fever, allergic rhinitis or eczema” in field 6152. Lastly, allergic disease cases were individuals who were classified as an “asthma case” and/or “hay fever/eczema case”, a total of 124,616 individuals.

To identify individuals who suffered from a single allergic disease, we had to classify hay fever and eczema status separately, which could not be determined using field 6152 *per se* (“Has a doctor ever told you that you have had any of the following conditions?”). This is because of the seven possible answers to this question, a single item covered the two different diseases, specifically the answer “Hayfever, allergic rhinitis or eczema”. To identify individuals who reported suffering specifically from hay fever, we instead considered information reported in the verbal interview (field 20002) and ICD10 codes (fields 41202 and 41204), as described above for asthma. This information was available for a subset of individuals who answered “Hayfever, allergic rhinitis or eczema” in field 6152. We then used the exact same approach (i.e. information from fields 20002, 41202 and 41204) to identify “eczema cases”.Using this approach, we were able to classify hay fever and eczema status separately, in addition to asthma, for 52,114 individuals. Of these, 975 suffered from all three diseases, 8,172 from two diseases and 42,967 from a single disease. Of the latter, 23,375 reported suffering from asthma only; 15,445 from hay fever only; and 4,147 from eczema only.

### Association analysis of the combined age-of-onset phenotype

We first performed multi-dimensional scaling (MDS) analysis of allele sharing to identify individuals who clustered closely to Europeans of the 1000 Genomes project, as described previously [22]. Of the 124,616 individuals with a phenotype available for analysis, 117,530 clustered within 5 standard deviations of the mean for the first and second MDS components estimated using individuals from the five European ancestry groups (CEU, GBR, FIN, IBS and TSI) of the 1000 Genomes project. As such, these individuals were considered to have European ancestry and were retained for analysis. We then excluded 400 individuals who: (i) had self-reported sex different from genetically-inferred sex; (ii) were outliers when considering genotype missing rates and/or genome-wide heterozygosity levels; (iii) had more than 10 third degree relatives or were excluded from kinship inference; and/or (iv) were not present in the imputed dataset released in July 2017. The first three criteria were assessed based on information included in the QC file ukb_sqc_v2.txt released by the UK Biobank study. After these exclusions, phenotype and genotype data were available for 117,130 individuals. Of these, 22,029 reported suffering from asthma only; 14,474 from hay fever only; and 3,939 from eczema only.

To maximize power, we did not select a subset of unrelated individuals for analysis but instead tested variants for association with age-of-onset using the linear mixed model implemented in BOLT-LMM [77], which includes a genetic relationship matrix (GRM) as a random effect in the model. The GRM was estimated based on 577,110 array SNPs obtained after quality control filters, namely a minor allele frequency (MAF) >1%, call rate >95% and Hardy-Weinberg equilibrium *P*-value >10^−6^. Age-of-onset was quantile-normalized prior to analysis; gender and an indicator of the genotyping array used were included as discrete covariates.

Of the 92 million variants with imputed data released by the UK Biobank, we analysed 7,647,814 variants that (i) had a MAF >1%; (ii) were imputed based on the Haplotype Reference Consortium panel; (iii) had matching alleles when compared to genotype data from the 1000 Genomes project; and (iv) had a unique reference sequence (rs) number and genomic position (based on hg19). We used a P-value threshold of 3x10^-8^ for genome-wide significance, as suggested for studies that analyse variants with a MAF >1% [78].

### Identification of variants with a statistically independent association with the combined age-of-onset phenotype

We used the approximate joint association analysis option of GCTA [79] to identify variants that remained associated with age-of-onset at a *P*<3x10^-8^ after accounting for the effects of nearby (<10 Mb) more strongly associated variants. In this analysis, LD was estimated based on a subset of 5,000 unrelated allergic disease cases from the UK Biobank study.

### Association analysis of age-of-onset in individuals suffering from a single allergic disease

To understand if variants discovered through the analysis of the combined age-of-onset phenotype were likely to influence the age-of-onset of the three individual diseases considered (asthma, hay fever and eczema), we performed association analyses in adult cases from the UK Biobank study who reported suffering from a single allergic disease, as described in detail previously [22].

Specifically, we tested the association between selected variants and age-of-onset separately in three non-overlapping groups of individuals who suffered from a single allergic disease: asthma only cases (*n* = 22,029), hay fever only cases (*n* = 14,474), and eczema only cases (*n* = 3,969). These sample sizes are smaller than indicated above (23,375, 15,445 and 4,147, respectively) because individuals of non-European ancestry were not included in the association analysis. For each SNP, we then compared the effect on age-of-onset (i.e. beta from the linear model) between individual diseases (i.e. asthma vs. hay fever, asthma vs. eczema and hay fever vs. eczema), using the formula z = sigma / SE_sigma, where sigma = beta_diseaseA–beta_diseaseB, and SE_sigma = sqrt(SE_beta_diseaseA^2 + SE_beta_diseaseB^2), which follows a normal distribution.

### Association between age-of-onset sentinel variants and allergic disease risk

We recently observed that many variants associated with the case-control status of allergic disease–defined by the presence of asthma, hay fever and/or eczema–were also associated with variation in age-of-onset amongst allergic disease cases [22]. We therefore reasoned that the reverse would also be likely: that many variants associated with variation in age-of-onset would be associated with disease liability, as suggested by Sarnowski et al. [29]. To test this, for each variant with a genome-wide significant association with age-of-onset in the analysis described above, we extracted association results from our recent GWAS of allergic disease [22]; after excluding overlapping samples (the UK Biobank study, *n* = 138,354), results were based on the analysis of 222,484 individuals, which included 137,883 cases with asthma and/or hay fever and/or eczema, and 84,601 disease-free controls. If a variant of interest was not directly tested in that GWAS, we extracted results for the most correlated proxy (with *r*^2^>0.8), if available.

### Genetic correlation between age-of-onset and disease case-control status

To understand the extent to which the same genetic variants contribute to variation in disease age-of-onset and disease liability, we applied LD-score regression [32] to the association summary statistics obtained in the GWAS of the combined age-of-onset phenotype carried out as part of this study (*n* = 117,130) and in the GWAS of a combined allergic disease case-control phenotype (*n* = 360,838) that we reported recently [22]. The genetic correlation between the two GWAS (which is not biased by sample overlap) was estimated based on 1.1 million HapMap 3 SNPs, all with a MAF>1%, as recommended previously [32].

To illustrate graphically the observed genetic correlation between disease liability and age-of-onset, we compared the age-of-onset between individuals with a high and low polygenic burden of allergic disease risk SNPs. Specifically, for each individual in the UK Biobank study, we calculated a polygenic risk score (PRS) as the weighted average of the number of disease-predisposing alleles across the 136 sentinel variants that we identified in our recent GWAS of allergic disease case-control status [22]. Weights for each SNP corresponded to the allelic effect (*i*.*e*. beta) reported in that GWAS [22]. We then restricted our analysis to individuals that reported suffering from a single disease, as described above: asthma only cases (*n* = 22,029), hay fever only cases (*n* = 14,474), and eczema only cases (*n* = 3,969). Within each of these case groups, we identified individuals with a PRS in the top 10% and bottom 10% for that group, and then compared the distribution of age-of-onset between the two PRS groups using descriptive statistics.

### Multivariate GWAS of allergic disease age-of-onset and case-control status

If two phenotypes have a genetic correlation that is significantly different from 0, then multivariate association analysis could potentially increase the power to identify variants that contribute to the heritability of both phenotypes, when compared to the alternative of testing each phenotype separately. Whether or not increased power is obtained with a multivariate test depends, for example, on the statistical approach used, the magnitude of the overall phenotypic correlation between the two phenotypes, as well as the magnitude and direction of the effect of the shared variant on the two phenotypes [33,80,81]. To analyse the joint association between single SNPs and allergic disease age-of-onset and case-control status, we used the recently described metaUSAT approach [33], for three main reasons. First, this approach is applicable to summary statistics from GWAS with unknown sample overlap. Second, it accommodates summary statistics from a mix of continuous and binary traits. And third, by combining two classes of tests (MANOVA and sum squared score tests), metaUSAT provides increased power over alternative methods when a SNP affects all phenotypes analysed [33]. MetaUSAT was applied to the summary statistics of the combined age-of-onset phenotype carried out as part of this study (*n* = 117,130) and the GWAS of a combined allergic disease case-control phenotype (*n* = 360,838) that we reported recently [22]. Because we were interested in identifying SNPs that were genome-wide significant (*i*.*e*. with *P*<3x10^-8^) in the multivariate but not in the separate univariate analyses, we first used approximate conditional analyses [79] to adjust the results from each GWAS for the effects of variants that had a statistically independent association at *P*<3x10^-8^ in the respective study (which were identified by the joint association analysis described above). After this adjustment, no single SNP had a *P*<3x10^-8^ in each of the two individual phenotype GWAS as expected. MetaUSAT was then applied to the adjusted GWAS; the Pearson correlation coefficient between z-scores of the two phenotypes was estimated based on SNPs not associated with either trait (i.e. with a *P*>0.05 for both).

A new multivariate test of association with similar properties to metaUSAT (*e*.*g*. applicable to summary statistics of GWAS with sample overlap), was reported just prior to the submission of this manuscript for publication–the MTAG approach [34]. We therefore tested if the multivariate associations discovered with metaUSAT were also supported by results from this different approach. For each SNP, instead of a multivariate P-value, MTAG returns an effect estimate (beta, SE and P-value) for each phenotype that incorporates information contained in the GWAS of the other phenotype. This increases the effective sample size of each analysis and so it improves the power to detect associations with variants that are shared between the two phenotypes [34].

### Sentinel variants not previously implicated in the aetiology of allergic disease

To determine if a sentinel variant was in LD with a SNP previously reported to associate with any allergic disease, we (i) identified all SNPs in LD (*r*^2^>0.05) with that sentinel variant, using genotype data from individuals of European descent from the 1000 Genomes Project [82] (*n* = 294, release 20130502_v5a); and (ii) determined if the sentinel variant or any of the correlated SNPs identified were reported to associate with any allergic disease (asthma, hay fever, eczema, food allergy or atopy) in the NHGRI-EBI GWAS catalog database [83], which was downloaded on the 10^th^ of January 2018. This catalogue was supplemented with recent studies not included at the time of submission of this manuscript^.^ [2, 31, 84].

### Predicting target genes of sentinel variants based on LD with eQTL and non-synonymous SNPs

We performed the following steps to identify genes for which variation in gene expression and/or protein sequence was associated with sentinel SNPs identified in the age-of-onset and multivariate GWAS.

First, we identified single nucleotide polymorphisms (SNPs) associated with variation in gene expression (i.e. expression quantitative trait loci (eQTL)) in published transcriptome studies of five broad tissue types relevant for allergic disease: individual immune cell types, lung, skin, spleen and whole-blood. We identified a total of 43 transcriptome studies reporting results from eQTL analyses in any one of those five tissue types (**S1 Table**). Some studies included multiple cell types, experimental conditions and/or eQTL types, resulting in a total of 101 separate eQTL datasets. For each eQTL dataset, we then (i) downloaded the original publication tables/files containing results for the eQTL reported; (ii) extracted the SNP identifier, gene name, association P-value and directional effect (if available; beta/z-score and effect allele); (iii) excluded eQTL located >1 Mb of the respective gene (i.e. *trans* eQTL), because often these are thought to be mediated by *cis* effects [85]; (iv) excluded eQTL with an association *P*>8.9x10^-10^, a conservative threshold that corrects for 55,765 genes (based on GENCODE v19), each tested for association with 1,000 SNPs (as suggested by others [86–88]); and (v) for each gene, used the—clump procedure in PLINK [89] to reduce the list of eQTL identified (which often included many correlated SNPs) to a set of ‘sentinel eQTL’, defined as the SNPs with strongest association with gene expression and in low LD (*r*^2^<0.05, linkage disequilibrium (LD) window of 2 Mb) with each other.

Second, we identified genes for which a sentinel eQTL reported in any of the 101 eQTL datasets described above was in high LD (*r*^2^>0.8) with a sentinel variant identified in the age-of-onset or multivariate GWAS. That is, we only considered genes for which there was high LD between a sentinel eQTL and a sentinel allergic disease variant, which reduces the chance of spurious co-localization.

Third, we used wANNOVAR [90] to identify genes containing non-synonymous SNPs amongst all variants in LD (*r*^2^>0.8) with any sentinel variants. SNPs in LD with sentinel variants were identified using genotype data from individuals of European descent from the 1000 Genomes Project [83] (*n* = 294, release 20130502_v5a).

### Association between sentinel variants and the risk of food allergy

Age-of-onset of food allergy was not available in the UK Biobank study and so it was not considered in our combined allergic disease phenotype. To partly address this limitation, we determined if variants identified in the age-of-onset or multivariate analysis also contribute to food allergy risk. Specifically, we extracted association results for selected variants from a GWAS of food allergy conducted in the GOFA cohort that we published recently [15], which included 497 children with a positive oral food challenge and 2,387 controls (the genomic inflation factor [λ] for this GWAS was 1.03). All cases were recruited at the time of diagnosis (mean age 2.1; 84% under the age of 4). The most common food allergy was observed with hen’s egg (58%), followed by peanut (44%) and cow’s milk (34%). To study the association between each variant and food allergy in adults, we extracted association results from a GWAS of food allergy conducted in the GERA cohort, which we also reported recently [16], including 1,104 adults with an ICD9 (i.e. hospital admission) code for food allergy and 23,945 controls. The genomic inflation factor for this GWAS was 1.01. Most cases were admitted to hospital because of an allergic reaction to fish/shellfish (58%), peanuts (16%), eggs (13%) or milk (8%).

This study was approved by the Human Ethics Committee of the QIMR Berghofer Medical Research Institute. Approval of individual studies has been reported in Ferreira et al. [22].

## Supporting information

S1 Data Potential impact of recall bias on SNP associations with age-of-onset of allergic disease (page 4–6) Potential impact on SNP associations of phenotypic misclassification amongst individuals reporting late onset disease (page 7–9). Ten genes that are predicted targets of novel allergic disease variants and that have a known function that is directly relevant to disease pathophysiology (page 10–15). Study Acknowledgments (page 16–19). References (page 20–23).
(DOC)

S1 Fig Distribution of allergic disease age-of-onset in UK Biobank participants (n = 117,130) who reported suffering from asthma and/or hay fever/eczema. (DOCX)

S2 Fig Distribution of the observed and expected association P-values for the GWAS of allergic disease age-of-onset in the UK Biobank study (n = 117,130). (DOCX)

S3 Fig Reliability of self-reported age-of-onset of asthma based on information provided by 1,650 UK Biobank participants at two time points. (DOCX)

S4 Fig Association between sentinel SNPs identified in the age-of-onset (Panel A) or multivariate GWAS (Panel B) and asthma age-of-onset in the subset of UK Biobank individuals who reported developing asthma as a child. (DOCX)

S5 Fig Association between sentinel SNPs identified in the age-of-onset (Panel A) or multivariate GWAS (Panel B) and hay fever age-of-onset in the subset of UK Biobank individuals who reported developing hay fever as a child. (DOCX)

S6 Fig Association between sentinel SNPs identified in the age-of-onset (Panel A) or multivariate GWAS (Panel B) and time to asthma onset in children of the ALSPAC study. (DOCX)

S7 Fig Summary of results from the GWAS of allergic disease age-of-onset in the UK Biobank study (n = 117,130), after adjusting single-SNP results for the effects of independently associated variants (i.e. with P<3x10^-8^ in the joint association analysis performed with GCTA. (DOCX)

S8 Fig Summary of results from the GWAS of allergic disease case-control status (n = 360,838) after adjusting single-SNP results for the effects of independently associated variants (i.e. with P<3x10^-8^ in the joint association analysis performed with GCTA). (DOCX)

S9 Fig Distribution of the observed and expected association P values for the GWAS of allergic disease age-of-onset in the UK Biobank study (n = 117,130), after adjusting single-SNP results for the effects of independently associated variants (i.e. with P<3x10^-8^ in the joint association analysis performed with GCTA. (DOCX)

S10 Fig Distribution of the observed and expected association P values for the GWAS of allergic disease case-control status (n = 360,838) after adjusting single-SNP results for the effects of independently associated variants (i.e. with P<3x10^-8^ in the joint association analysis performed with GCTA. (DOCX)

S11 Fig Distribution of the observed and expected association P values obtained in the multivariate analysis of the GWAS of allergic disease risk (n = 360,838) and GWAS of allergic disease age-of-onset (n = 117,130). (DOCX)

S12 Fig Association between sentinel SNPs identified in the age-of-onset (Panel A) or multivariate GWAS (Panel B) and food allergy in children. (DOCX)

S1 Table Genome-wide association studies of gene expression levels queried to identify expression quantitative trait loci (i.e. eQTL). (XLS)

S2 Table Descriptive statistics for the 117,130 allergic disease cases from the UK Biobank included in this study. (XLS)

S3 Table Results from joint association analysis performed with GCTA ^79^. (XLS)

S4 Table Variants associated with allergic disease age-of-onset at a P<3x10^-8^ in the joint association analysis but not in the original analysis. (XLS)

S5 Table Association with allergic disease age-of-onset for variants previously reported in the literature. (XLS)

S6 Table Association between the 50 sentinel variants and age-of-onset of asthma, hay fever and eczema separately. (XLS)

S7 Table Sentinel SNPs in LD (r^2^>0.05) with variants previously reported to associate with at least one allergic disease. (XLS)

S8 Table Results from the MTAG multivariate approach for the sentinel variants identified in the metaUSAT multivariate analysis of allergic-disease age-of-onset and allergic disease case-control status. (XLS)

S9 Table Sentinel eQTL in LD (r^2^>0.5) with sentinel variants associated with allergic disease age-of-onset. (XLS)

S10 Table Sentinel eQTL in LD (r^2^>0.8) with sentinel variants jointly associated with allergic disease age-of-onset and allergic-disease risk. (XLS)

S11 Table Non-synonymous SNPs in LD (r^2^>0.8) with sentinel variants associated with allergic disease. (XLS)

S12 Table Two independent associations with self-reported food allergy case-control status at a P<3x10^-8^. (XLS)

S13 Table Association between the 76 sentinel SNPs and self-reported food allergy case-control status. (XLS)

S14 Table Results of replication study of age-of-onset GWAS in ALSPAC. (XLS)
