# Supplementary material for: Age-of-onset information helps identify 76 genetic variants associated with allergic disease
Source: PLoS Genet. 2020 Jun 30;16(6):e1008725. doi: 10.1371/journal.pgen.1008725 (PMC7367489; doi:10.1371/journal.pgen.1008725)
Supplement: S1 Data — Ten genes that are predicted targets of novel allergic disease variants and that have a known function that is directly relevant to disease pathophysiology (page 10–15). Study Acknowledgments (page 16–19). References (page 20–23). (DOC) [file pgen.1008725.s001.doc]

**Age-of-onset information helps identify 76 genetic variants associated with allergic disease**

**SUPPLEMENTARY INFORMATION**

Manuel AR Ferreira^1^, Judith M Vonk^2^, Hansjörg Baurecht^3^, Ingo Marenholz^4,5^, Chao Tian^6^, Joshua D Hoffman^7^, Quinta Helmer^8^, Annika Tillander^9^, Vilhelmina Ullemar^9^, Yi Lu^9^, Sarah Grosche^4,5^, Franz Rüschendorf^4^, Raquel Granell^10^, Ben M Brumpton^10,11,12^, Lars G Fritsche^11,13^, Laxmi Bhatta^11^, Maiken E Gabrielsen^11^, Jonas B Nielsen^14,15^, Wei Zhou^15^, Kristian Hveem^11^, Arnulf Langhammer^16^, Oddgeir L Holmen^11^, Mari Løset^11,17^, Gonçalo R Abecasis^11,13^, Cristen J Willer^13,14,15^, Nima C. Emami^18,19^, Taylor B. Cavazos^18^, John S. Witte^18,19,20,21^, Agnieszka Szwajda^22^, the 23andMe Research Team^6^, collaborators of the SHARE study^23^, David A Hinds^6^, Norbert Hübner^4^, Stephan Weidinger^3^, Patrik KE Magnusson^9^, Eric Jorgenson^24^, Robert Karlsson^9^, Lavinia Paternoster^10^, Dorret I Boomsma^8^, Catarina Almqvist^9,25^, Young-Ae Lee^4,5^ and Gerard H Koppelman^26^

**Collaborators of the SHARE study**

Jorge Esparza-Gordillo^1,2,3^, Oliver Hummel^1^, Sarah Grosche^1,2^, John S Witte^4^, Jouke-Jan Hottenga^5^, Gonneke Willemsen^5^, Elke Rodríguez^9^, Melanie Hotze^9^, Andre Franke^10^, Melanie C Matheson^11^, Shyamali C Dharmage^11^, Andreas Arnold^12^, Georg Homuth^13^, Carsten O Schmidt^14^, Philip J Thompson^15^, Nicholas G Martin^16^, David L Duffy^16^, Natalija Novak^17^, Holger Schulz^18,19^, Stefan Karrasch^18,20^, Christian Gieger^21^, Konstantin Strauch^22,23^ and Ronald B Melles^24^

1 Max Delbrück Center (MDC) for Molecular Medicine, Berlin, Germany

2 Clinic for Pediatric Allergy, Experimental and Clinical Research Center of Charité Universitätsmedizin Berlin and Max Delbrück Center, Berlin, Germany

3 Current address: GlaxoSmithKline, Stevenage, UK

4 Epidemiology and Biostatistics, University of California San Francisco, San Francisco, California, USA

5 Department Biological Psychology, Netherlands Twin Register , Vrije University, Amsterdam, The Netherlands

9 Department of Dermatology, Allergology and Venereology, University Hospital Schleswig-Holstein, Campus Kiel, Kiel, Germany

10 Institute of Clinical Molecular Biology, Christian Albrechts University of Kiel, Kiel, Germany

11 Melbourne School of Population and Global Health, University of Melbourne, Melbourne, Australia

12 Clinic and Polyclinic of Dermatology, University Medicine Greifswald, Greifswald, Germany

13 Department of Functional Genomics, Interfaculty Institute for Genetics and Functional Genomics, University Medicine and Ernst-Moritz-Arndt-University Greifswald, Greifswald, Germany

14 Institute for Community Medicine, Study of Health in Pomerania/KEF, University Medicine Greifswald, Greifswald, Germany

15 Institute for Respiratory Health, Harry Perkins Institute of Medical Research, University of Western Australia, Nedlands, Australia

16 Genetics and Computational Biology, QIMR Berghofer Medical Research Institute, Brisbane, Australia

17 Department of Dermatology and Allergology, University-Hospital Bonn, Bonn, Germany

18 Institute of Epidemiology I, Helmholtz Zentrum Munchen - German Research Center for Environmental Health , Neuherberg, Germany

19 Comprehensive Pneumology Center Munich (CPC-M), Member of the German Center for Lung Research, Munich, Germany

20 Institute and Outpatient Clinic for Occupational, Social and Environmental Medicine, Ludwig-Maximilians-Universität, Munich, Germany

21 Research Unit of Molecular Epidemiology and Institute of Epidemiology II,, Helmholtz Zentrum Munchen - German Research Center for Environmental Health , Neuherberg, Germany

22 Institute of Genetic Epidemiology, Helmholtz Zentrum Munchen - German Research Center for Environmental Health , Neuherberg, Germany

23 Chair of Genetic Epidemiology, IBE, Faculty of Medicine, LMU Munich, Germany

24 Division of Research, Kaiser Permanente Northern California, Oakland, California, USA

**Potential impact of recall bias on SNP associations with age-of-onset of allergic disease**

All UK Biobank participants included in our analyses were adults (aged 38 to 70) at the time of data collection. As such, we were concerned that recall bias might have affected the reported age-of-onset and so potentially the robustness of our findings. For example, older participants at the time of data collection might have recalled early onset of disease less reliably and/or less frequently than younger participants. To address this potential limitation, we (1) asked if allergic disease age-of-onset reported by UK Biobank participants was reliable and indeed likely to be affected by recall bias; and (2) performed two sets of association analyses for which recall bias was not a major concern and tested if results were consistent with our findings for the 50 sentinel variants described above. For simplicity and to help interpret results, we focused these analyses on age-of-onset of asthma (instead of allergic disease).

*Reliability of self-reported asthma age-of-onset in UK Biobank participants and evidence of recall bias.* We compared the reported age at which asthma was diagnosed by a doctor (field 3786: “What was your age when the asthma was first diagnosed?”) between two separate surveys, the initial assessment visit (completed between 2006 and 2010; instance 0, encoded in variable f.3786.0.0) and the first repeat assessment visit (completed between 2012 and 2013; instance 1, encoded in variable f.3786.1.0). A total of 1,650 individuals provided a valid age-of-onset (>=1) at these two time points, with a strong correlation between the two (*r*=0.93, *P*<2x10^-16^; **Supplementary Figure 3**). The two reported ages were exactly the same for 35% of individuals, within 2 years for 68%, and within 5 years for 86%. Thus, asthma age-of-onset information provided by most individuals had an effective resolution of at least 5 years. For an additional ~10% of individuals, age-of-onset information was less reliable (resolution of ~10-15 years), but still likely to be informative to detect SNP effects on a broader age scale, for example, comparing childhood- versus adult-onset asthma. Lastly, for <5% of individuals self-reported asthma age-of-onset was not sufficiently reliable (>15 years difference between the two visits), and so their inclusion in our association analyses might have decreased power to detect true SNP associations with age-of-onset.

We also tested if the (absolute) difference in age-of-onset reported at the two surveys described above was correlated with age at enrollment. We found a relatively modest but significant positive correlation (Pearson correlation 0.08, *P*=0.002), which indicates that older participants were slightly more likely to report a more unreliable age-of-onset. For example, 3.9% of participants aged 60 or older at enrolment (n=633) reported an age-of-onset that differed by >15 years between the two visits, compared to 1.4% of participants aged 50 or younger (n=422).

To determine if recall bias was likely to have affected the asthma age-of-onset reported by UK Biobank participants, we tested if childhood-onset asthma, defined by disease onset at or before age 19, was less likely to be reported by older participants. When considering all asthmatics with available information on age-of-onset (n=51,679), we found that age at enrolment was strongly negatively associated with a report of childhood-onset asthma (odds ratio of 0.99 per year, SE=0.0002, *P*<2x10^-16^). For example, participants aged 60 or older (n=20,457) reported childhood-onset asthma 2-fold less frequently when compared to participants 50 or younger (n=15,523). It is likely that these results are explained by recall bias, that is, older participants did not recall childhood-onset asthma as accurately as younger participants. If this was the case, then misclassification of age-of-onset in older participants might have decreased the power to detect true associations with age-of-onset in our analyses. On the other hand, it is also possible that the increase in asthma prevalence documented in recent decades might have contributed, at least partly, to the higher frequency of childhood-onset asthma reported by younger participants.

*Association between sentinel variants and age-of-onset in a subset of individuals who reported developing asthma as a child.* We performed two sets of association analyses for which the effect of recall bias on self-reported age-of-onset was minimized and so unlikely to be a major concern. In the first analysis, described in this section, we tested each of the 50 sentinel variants for association with asthma age-of-onset in a subset of 13,962 individuals from the UK Biobank study who reported developing asthma as a child (age 0 to 19). Results for asthma onset in this smaller but more homogeneous case group were highly consistent with results for allergic disease onset obtained when considering all 117,130 cases (**Supplementary Figure 4A**). Similar results were observed for the 26 sentinel variants identified in the multivariate GWAS (**Supplementary Figure 4B)**, as well as when considering hay fever age-of-onset in individuals who were first diagnosed with hay fever as a child (*n*=10,840; **Supplementary Figure 5**). Hay fever age-of-onset was determined based on the UK Biobank field 22146: “age you were first diagnosed [with hayfever or allergic rhinitis] by a doctor”. This analysis was not performed for eczema onset due to small sample size.

*Association between sentinel variants and age-of-onset in children from the ALSPAC study.* In the second analysis, we tested if a consistent association with the 50 sentinel SNPs was observed when analyzing time to asthma onset in children in the independent ALSPAC study (*n*=3,420, mean age 13.9), as reported in detail previously^1^. Forty SNPs were tested in that study (either directly or via a proxy with *r*^2^>0.8), of which 34 had a directionally consistent association (**Supplementary Figure 6A**), a statistically significant enrichment over the 50% null expectation (binomial *P*=7x10^-7^). Similar results were observed for the 26 sentinel variants identified in the multivariate GWAS (**Supplementary Figure 6B)**.

Therefore, the 76 sentinel variants reported in our study show a consistent pattern of association with asthma age-of-onset in two analyses for which recall bias was not a major concern.

**Potential impact on SNP associations of phenotypic misclassification amongst individuals reporting late onset disease**

Another related consideration is that, proportionally, there were many individuals in the UK Biobank study with a reported late onset of allergic disease (*e.g.* 41% of asthmatics had an onset at age 40 or later), which again might reflect recall bias but also potentially phenotypic misclassification. We were specifically concerned about the possibility that many cases who reported late onset of allergic disease actually did not truly suffer from allergic disease, instead having been misclassified by a doctor, or incorrectly reported suffering from asthma, hay fever and/or eczema in the UK Biobank survey. We performed two sets of analyses to address this possibility, as described below. Again, we focused these analyses on asthma age-of-onset for simplicity and to facilitate interpretation.

*Genetic correlation between adult-onset asthma in the UK Biobank study and previously published asthma GWAS.* We reasoned that if many cases with adult-onset asthma in the UK Biobank study did not truly suffer from asthma (i.e. were misclassified), then we would not expect to observe a close agreement in SNP associations between a GWAS of adult-onset asthma in the UK Biobank study and previously published GWAS of asthma.

UK Biobank individuals with asthma onset as an adult were identified based on (i) a report of asthma in field 6152 (self-reported medical conditions); and (ii) age-of-onset of asthma >=40 reported in field 3786. We used a cut-off of 40 years of age to minimize the overlap with UK Biobank cases who reported developing asthma as a child or young adult. A total of 19,332 cases were identified after applying the exclusion filters described in the Methods section (*e.g.* ancestry outliers). Controls were those who did not report suffering from asthma in field 6152, a total of 406,724 individuals. Approximately 9 million SNPs were tested for association with case-control status using BOLT-LMM, as described in the Methods section; the LD-score intercept of this analysis was 1.00.

Next, we used the LD-score regression approach ^2^ to determine the extent to which SNPs associated with adult-onset asthma in the UK Biobank study were collectively also associated with asthma risk in previous GWAS. Based on results from HapMap3 SNPs, we observed a high genetic correlation between the GWAS of adult-onset asthma in the UK Biobank study and the GWAS of asthma performed by the GABRIEL consortium ^3^ (*r*_g_=0.62, *P*=7x10^-11^), which included 10,365 cases (65% with pediatric asthma, i.e. onset <=16 years of age) and 16,110 controls, tested for association with 446K array SNPs. A similarly high genetic correlation was obtained with the asthma GWAS reported more recently by Demenais et al. ^4^ (*r*_g_=0.66, *P*=8x10^-25^), which included 19,954 cases (36% with pediatric asthma) and 107,715 controls tested for association with ~2 million HapMap SNPs. From these results we conclude that, even if present, phenotypic misclassification amongst individuals with self-reported adult-onset asthma in the UK Biobank study was not sufficiently common to prevent the identification of true-positive SNP associations with asthma.

*Genetic correlation between adult-onset asthma in the UK Biobank study and adult-onset asthma in the HUNT study.* A limitation of the previous analysis was that the two published asthma GWAS considered included a mixture of both childhood- and adult-onset asthma cases, which might have confounded the observed genetic correlations. To address this possibility, we performed a GWAS of adult-onset asthma in the Norwegian HUNT study and then estimated its genetic correlation with the GWAS of adult-onset asthma in the UK Biobank study.

To minimize the impact of recall bias, we used longitudinal information on asthma status (rather than self-reported age-of-onset) collected in two HUNT surveys conducted 11 years apart (on average), as described recently^5^. Briefly, we identified 1,365 cases who answered “No” to the question “Do you have or have you had asthma?” at the HUNT2 survey (mean age 47.8, SD 13.5, range 19.2-79.5) but then answered “Yes” to the same question at the HUNT3 survey (mean age 59.0, SD 13.4, range 30.5- 89.9). Asthma-free controls were those who answered “No” to this question both at the HUNT2 (mean age 46.9, SD 13.3, range 19.2-89.8) and HUNT3 (mean age 58.1, SD 13.3, range 29.3-100.8) surveys, a total of 31,673 individuals. A total of 8,464,124 SNPs that were common (MAF>0.05), well imputed (info score >0.3) and present in the 1000 Genomes Project were tested for association with adult-onset asthma using SAIGE; the LD-score intercept of this analysis was 1.00.

Using the LD-score regression approach ^2^, we observed a significant genetic correlation between the HUNT and UK Biobank analyses of adult-onset asthma: *r_g_*=0.69 (SE=0.269, *P*=0.011), consistent with the estimates obtained when considering the GABRIEL and Demenais et al. asthma GWAS. These results further support the notion that misclassification of disease status is unlikely to have been a major contributing factor to the disproportionally large number of individuals in the UK Biobank study with a reported late onset of allergic disease.

**Ten genes that are predicted targets of novel allergic disease variants and that have a known function that is directly relevant to disease pathophysiology**

A disintegrin and metalloproteinase 15 (*ADAM15*) was identified as a likely target of a sentinel variant (rs4971089) identified in the age-of-onset GWAS. Our directional effect analysis indicated that the rs4971089:A allele that delays the onset of allergic disease was associated with increased *ADAM15* expression in blood. This protective effect of genetically-determined increased gene expression on disease onset is consistent with the anti-inflammatory role described for this molecule by *in vitro* studies. For example, ADAM15 was found to cleave the toll like receptor (TLR) adaptor molecule TRIF from the cell membrane, thereby reducing the production of pro-inflammatory mediators induced by TLR3 or TLR4 activation ^6^. ADAM15 also cleaves the low affinity IgE receptor (FCER2 or CD23) ^7^, suggesting another mechanism by which the rs4971089:A allele might lead to a delayed onset of allergic disease. In contrast to these findings, Sun et al. reported that *Adam15* knock-out mice had reduced LPS-induced inflammation ^8^. In the GTEx study ^9^, *ADAM15* is most highly expressed in the skin, with high expression also detected in lung; murine T cells have also been reported to express *ADAM15* ^10^.

Fos-like antigen 2 (*FOSL2*), which was also identified as a target of an age-of-onset sentinel variant, is part of the transcription factor complex AP-1 ^11^. The expression of *FOSL2* is induced by IL-2 in activated CD4+ T-cells through a STAT5-dependent mechanism ^12^, and is up-regulated during early Th2 cell polarization ^13^. *FOSL2* is also involved in B cell and epidermal differentiation ^14, 15^, and has a critical yet complex role in Th17 differentiation and function ^16^.

Tripartite motif-containing protein 8 (*TRIM8*) was one of five genes predicted as targets of rs12572775, which is located in the SUFU gene on chromosome 10q24. TRIM8 targets TRIF (as noted for ADAM15), and so TLR3- and TLR4-mediated innate immune responses are up-regulated in Trim8 knock-out mice ^17^. The latter findings support a protective role for increased *TRIM8* expression in allergic disease risk. Consistent with this possibility, we observed that the rs12572775:T allele that delayed age-of-onset was associated with increased expression of *TRIM8*.

The bone morphogenetic protein receptor type II (*BMPR2*) gene was one of two predicted targets of sentinel variant rs72926957. Mutations in BMPR2, which encodes a receptor that binds proteins of the TGF-beta superfamily (e.g. BMP4 and BMP7)^18^, cause pulmonary arterial hypertension^19^, partly by inhibiting Smad-mediated signaling pathways ^20^. Of more obvious relevance to allergic disease, BMPR2 is expressed in the thymus and BMP4 treatment inhibits the differentiation of CD4-CD8- cells long the T cell lineage ^21^. The second predicted target of rs72926957 was the family with sequence similarity 117 member B (*FAM117B*); this target was predicted based on correlated (*r*^2^>0.9) sentinel eQTLs identified in whole-blood as well as in multiple immune cell subsets (e.g. monocytes and neutrophils). To our knowledge, the function of *FAM117B* is unknown; relevant tissues with highest expression in the GTEx study included skin and spleen. Of note, variants in high LD (*r*^2^>0.9) with rs72926957 have been reported to associate with total cholesterol ^22^ and sarcoidosis ^23^.

The CD200 receptor 1 gene (*CD200R1*) was found to contain four non-synonymous variants and one eQTL in complete LD (*r*^2^=1) with a sentinel variant identified in the multivariate GWAS (rs9870568). These results indicate that both variation in CD200R1 protein sequence and mRNA levels influence allergic disease risk and age-of-onset. *CD200R1* encodes a surface glycoprotein that interacts with CD200 ^24^, being highly expressed in dendritic cells polarized Th2 cells, mast cells, neutrophils and basophils ^25^. Engagement of CD200R1 by CD200 has been shown to suppress the activation of various immune cells, including macrophages ^26^, mast cells ^27^, monocytes ^28^ and dendritic cells ^29^. Given these findings, drugs that activate CD200R1 might be expected to increase immune suppression and so improve allergic disease symptoms, as suggested recently in experimental asthma ^30^. In contrast to these studies, however, our results indicate that genetically determined lower *CD200R1* expression – which is expected to result in increased immune activation – was associated with lower disease risk and delayed age-of-onset, the opposite effect of that suggested by functional studies. There are a number of possible explanations for this apparent discrepancy. First, the sentinel variant might affect allergic disease pathophysiology because of its effect on CD200R1 protein sequence and not gene transcription levels. Of interest, the four non-synonymous variants that were in LD with the sentinel variant affected amino-acids located in the extracellular (three variants) or cytoplasmic (one variant) domains. Second, a soluble form of CD200R1 has recently been recognized ^31^, and so it is possible that the sentinel variant has opposing effects on CD200R1 mRNA and soluble CD200R1 levels, as described for example for IL6R eQTL ^32^. Third, lower *CD200R1* expression might result in stronger anti-viral responses in early life ^33^, which could potentially decrease allergic disease risk and delayed its onset. Lastly, CD200R1 might not be the actual target of the allergy sentinel variant identified. We note that other relevant genes are located nearby (<1 Mb), including *CD200* and *CD200R1L*.

The protein kinase C theta gene (*PRKCQ)* encodes a protein kinase with a critical role in T-cell activation ^34, 35^. In the context of allergic disease, *PRKCQ* is required for the development of Th2 responses in the lung ^36^, at least partly through its role in the activation of type 2 innate lymphoid cells ^37^. *PRKCQ* also promotes Th17 differentiation ^38^. In contrast, *PRKCQ* knock-out mice have reduced numbers of Treg cells ^39^, suggesting that this gene might have both pro- and anti-inflammatory roles in allergic disease. Of note, results from our eQTL analysis indicate that increased *PRKCQ* expression in leukocytes is associated with decreased allergic disease risk and delayed onset.

The nucleotide-binding oligomerization domain protein 2 gene (*NOD2*) was the first genetic risk factor identified for Crohn’s disease ^40, 41^. To our knowledge, Sarnowski et al. ^1^ were the first to report an association between variants near *NOD2* and allergic disease, specifically variation in asthma age-of-onset. However, as indicated above, we did not find any evidence for association with age-of-onset in our analyses with the specific variant reported by Sarnowski et al. Instead, we identified two independent associations in the multivariate GWAS that point to *NOD2* as an important player in allergic disease pathophysiology. Both variants are relatively uncommon, with a minor allele frequency <5%. The first association was with rs2066844, a non-synonymous variant in *NOD2* (R702W) previously reported to associate with Crohn’s disease ^42^: the directional effect was the same in both diseases, with the minor allele (T, corresponding to 702W, which is predicted to inhibit *NOD2* signaling) associated with increasing disease risk (and decreasing age-of-onset of allergic disease). The second association was with rs8056255, an intergenic variant that is in LD with a sentinel eQTL for *NOD2* in whole-blood. Based on results for this eQTL, decreased *NOD2* expression was associated with increase disease risk and early age-of-onset. NOD2 encodes an intracellular pattern recognition receptor that binds bacterial peptidoglycans ^43^. Activation of NOD2 then promotes host defense through the production of inflammatory cytokines ^44, 45^ and anti-microbial peptides ^46^. On the other hand, NOD2 is also essential for host defense against viral infection. For example, Sabbah et al. ^47^ reported that Nod2-deficient mice infected with respiratory syncytial virus (RSV) had lower IFN-beta production in the respiratory tract and higher viral titers than wild-type mice. Thus, it is possible that variants that inhibit NOD2 signaling result in an increased risk of allergic disease because of their detrimental effect on anti-viral immunity.

The mothers against decapentaplegic drosophila homolog 4 gene (*SMAD4*) encodes a central mediator of TGF-beta signaling, transducing signals from the activated TGF-beta receptor into the nucleus^48^. Of relevance to allergic disease, Smad4^-/-^ murine T cells have increased production of Th2 cytokines ^49^, impaired polarization into Tregs ^50^ and can differentiate into Th17 cells in the absence of TGF-beta signaling ^51^. In contrast, Smad4^-/-^ murine T cells have decreased TGF-beta-induced expression of selectin ligands ^52^, which are required for T cells to migrate to inflamed tissues, and impaired expression of IL-9, a pro-allergic cytokine ^53^. Given these opposing effects of SMAD4 inhibition on disease-relevant cellular mechanisms, it is not clear if drug development for allergic disease should focus on drugs that decrease or increase SMAD4 expression/function. We suggest that the former and not the latter are likely to attenuate disease symptoms, given our observation that genetically-determined lower SMAD4 expression in blood was associated with reduced risk and increased age-of-onset of allergic disease.

The ATP-binding cassette subfamily A member 7 gene (*ABCA7*) encodes a transported protein that moves lipids across membranes ^54^, being highly expressed in leukocytes, thymus and spleen ^55^. ABCA7 has been shown to enhance phagocytosis of apoptotic cells by macrophages ^56^ and NKT cell development and function ^57^. It has also been suggested to play a role in the terminal differentiation of keratinocytes ^58^. Loss-of-function variants in ABCA7 increase the risk of Alzheimer’s disease ^59^, as do nearby common variants ^60^. The variant identified in our multivariate GWAS is not in LD with the latter (*r*^2^=0.02) and so does not represent a risk factor shared between allergic disease and Alzheimer’s disease.

The ubiquitin-conjugating enzyme E2L 3 gene (*UBE2L3*) encodes an essential component of the post-translational protein ubiquitination pathway, which plays a major role in the regulation of inflammatory responses ^61^. UBE2L3 has been suggested to promote TNF-alpha induced IL6 and IL8 expression, through its effect on NF-kB signaling ^62^. Increased NF-kB translocation has been suggested to underlie the association between alleles that increase the expression of UBE2L3 and the risk of multiple auto-immune diseases, including systemic lupus erythematosus ^63^. In contrast, UBE2L3 has also been reported to negatively regulate T cell activation ^64^ and to act as an adaptor for NDFIP1 ^65^, another risk gene for allergic disease ^66^ that is a negative regulator of pro-inflammatory cytokines ^67, 68^. UBE2L3 was also recently reported to promote the production of pro-interleukin 1 beta ^69^. As observed for auto-immune diseases, the rs5754217:T allele that was associated with increased disease risk was in LD with alleles that increased *UBE2L3* expression in multiple tissues or cell types, including whole-blood and leukocytes.

**Acknowledgments**

23andMe: We would like to thank the research participants and employees of 23andMe for making this work possible. We particularly thank the following members of the 23andMe Research Team: Michelle Agee, Babak Alipanahi, Adam Auton, Robert K. Bell, Katarzyna Bryc, Sarah L. Elson, Pierre Fontanillas, Nicholas A. Furlotte, Bethann S. Hromatka, Karen E. Huber, Aaron Kleinman, Nadia K. Litterman, Jennifer C. McCreight, Matthew H. McIntyre, Joanna L. Mountain, Elizabeth S. Noblin, Carrie A.M. Northover, Steven J. Pitts, J. Fah Sathirapongsasuti, Olga V. Sazonova, Janie F. Shelton, Suyash Shringarpure, Joyce Y. Tung, Vladimir Vacic, and Catherine H. Wilson.

LifeLines: The LifeLines Biobank initiative has been made possible by funds from FES (Fonds Economische Structuurversterking), SNN (Samenwerkingsverband Noord Nederland) and REP (Ruimtelijk Economisch Programma). The authors wish to acknowledge the services of the LifeLines Cohort Study, the contributing research centres delivering data to LifeLines, and all the study participants.

SALTY/TWINGENE/CATSS: We acknowledge the Swedish Twin Registry for access to data. The Swedish Twin Registry is managed by Karolinska Institutet and receives funding from the Swedish Research Council under the grant no 2017-00641.

ALSPAC: Avon Longitudinal Studies of Parents and Children. Pregnant women resident in Avon, UK with expected dates of delivery 1st April 1991 to 31st December 1992 were invited to take part in the study. The initial number of pregnancies enrolled is 14,541 (for these at least one questionnaire has been returned or a “Children in Focus” clinic had been attended by 19/07/99). Of these initial pregnancies, there was a total of 14,676 foetuses, resulting in 14,062 live births and 13,988 children who were alive at 1 year of age.

When the oldest children were approximately 7 years of age, an attempt was made to bolster the initial sample with eligible cases who had failed to join the study originally. As a result, when considering variables collected from the age of seven onwards (and potentially abstracted from obstetric notes) there are data available for more than the 14,541 pregnancies mentioned above. The number of new pregnancies not in the initial sample (known as Phase I enrolment) that are currently represented on the built files and reflecting enrolment status at the age of 24 is 913 (456, 262 and 195 recruited during Phases II, III and IV respectively), resulting in an additional 913 children being enrolled. The phases of enrolment are described in more detail in the cohort profile paper and its update. The total sample size for analyses using any data collected after the age of seven is therefore 15,454 pregnancies, resulting in 15,589 foetuses. Of these 14,901 were alive at 1 year of age.

A 10% sample of the ALSPAC cohort, known as the Children in Focus (CiF) group, attended clinics at the University of Bristol at various time intervals between 4 to 61 months of age. The CiF group were chosen at random from the last 6 months of ALSPAC births (1432 families attended at least one clinic). Excluded were those mothers who had moved out of the area or were lost to follow-up, and those partaking in another study of infant development in Avon.

Ethical approval for the study was obtained from the ALSPAC Ethics and Law Committee and the Local Research Ethics Committees. Further details are available in the cohort profile paper ^70-71^ and the study website contains details of all the data that is available through a fully searchable data dictionary and variable search tool" http://www.bristol.ac.uk/alspac/researchers/our-data/.

The UK Medical Research Council and Wellcome (Grant ref: 102215/2/13/2) and the University of Bristol provide core support for ALSPAC. GWAS data was generated by Sample Logistics and Genotyping Facilities at Wellcome Sanger Institute and LabCorp (Laboratory Corporation of America) using support from 23andMe. A comprehensive list of grants funding is available on the ALSPAC website (http://www.bristol.ac.uk/alspac/external/documents/grant-acknowledgements.pdf);

We are extremely grateful to all the families who took part in this study, the midwives for their help in recruiting them, and the whole ALSPAC team, which includes interviewers, computer and laboratory technicians, clerical workers, research scientists, volunteers, managers, receptionists and nurses.

AAGC: The AAGC was funded by a grant from the NHMRC (project ID 613627).

GENEVA: The project received infrastructure support through the DFG Clusters of Excellence “Inflammation at Interfaces” (grants EXC306 and EXC306/2), and was supported by the German Federal Ministry of Education and Research (BMBF) within the framework of the e:Med research and funding concept (sysINFLAME, grant # 01ZX1306A), and the PopGen 2.0 network (01EY1103). The KORA study was initiated and financed by the Helmholtz Zentrum München – German Research Center for Environmental Health, which is funded by the German Federal Ministry of Education and Research (BMBF) and by the State of Bavaria. Furthermore, KORA research was supported within the Munich Center of Health Sciences (MC-Health), Ludwig-Maximilians-Universität, as part of LMUinnovativ.

NTR: This study was supported by multiple grants from the Netherlands Organization for Scientific Research (NWO: 016-115-035, 463-06-001, 451- 04-034); ZonMW (31160008, 911-09-032); and NWO 480-15-001/674: Netherlands Twin Registry Repository: researching the interplay between genome and environment; The Amsterdam Public Health Institute (APH) and Neuroscience Campus Amsterdam (NCA); Biomolecular Resources Research Infrastructure (BBMRI–NL, 184.021.007), European Research Council (ERC-230374); Genotyping was made possible by grants from NWO/SPI 56-464-14192, Genetic Association Information Network (GAIN) of the Foundation for the National Institutes of Health, Rutgers University Cell and DNA Repository (NIMH U24 MH068457-06), the Avera Institute, Sioux Falls (USA) and the National Institutes of Health (NIH R01 HD042157-01A1, MH081802, Grand Opportunity grants 1RC2 MH089951 and 1RC2 MH089995).

GENUFAD-SHIP-1.We thank all individuals and families for their participation in this study. We thank all physicians and nurses involved in patient recruitment for their valuable contribution to the study. We are grateful to the laboratory technicians C. Flachmeier and T. Thuss for their work. The study was funded by the German Ministry of Education and Research (BMBF) through the Clinical Research Group for Allergy at Charité Berlin, the National Genome Research Network (NGFN). The SHIP authors are grateful to Mario Stanke for the opportunity to use his server cluster for SNP imputation. We thank all staff members and participants of the SHIP studies, as well as all of the genotyping staff for generating the SHIP SNP data set. SHIP is part of the Community Medicine Research net of the University of Greifswald, Germany, which is funded by the Federal Ministry of Education and Research (grants no. 01ZZ9603, 01ZZ0103, and 01ZZ0403), the Ministry of Cultural Affairs as well as the Social Ministry of the Federal State of Mecklenburg-West Pomerania, and the network ‘Greifswald Approach to Individualized Medicine (GANI_MED)’ funded by the Federal Ministry of Education and Research (grant 03IS2061A). Genome-wide data were supported by the Federal Ministry of Education and Research (grant 03ZIK012) and a joint grant from Siemens Healthcare, Erlangen, Germany, and the Federal State of Mecklenburg–West Pomerania. The University of Greifswald is a member of the 'Center of Knowledge Interchange' program of the Siemens AG and the Caché Campus program of the InterSystems GmbH.

GENUFAD-SHIP-2. We thank all individuals and families for their participation in this study. We thank all physicians and nurses involved in patient recruitment for their valuable contribution to the study. We are grateful to the laboratory technicians C. Flachmeier and T. Thuss for their work. The study was funded by the German Ministry of Education and Research (BMBF) through the Clinical Research Group for Allergy at Charité Berlin, the National Genome Research Network (NGFN). The SHIP authors are grateful to Mario Stanke for the opportunity to use his server cluster for SNP imputation. We thank all staff members and participants of the SHIP studies, as well as all of the genotyping staff for generating the SHIP SNP data set. SHIP is part of the Community Medicine Research net of the University of Greifswald, Germany, which is funded by the Federal Ministry of Education and Research (grants no. 01ZZ9603, 01ZZ0103, and 01ZZ0403), the Ministry of Cultural Affairs as well as the Social Ministry of the Federal State of Mecklenburg-West Pomerania, and the network ‘Greifswald Approach to Individualized Medicine (GANI_MED)’ funded by the Federal Ministry of Education and Research (grant 03IS2061A). Genome-wide data were supported by the Federal Ministry of Education and Research (grant 03ZIK012) and a joint grant from Siemens Healthcare, Erlangen, Germany, and the Federal State of Mecklenburg–West Pomerania. The University of Greifswald is a member of the 'Center of Knowledge Interchange' program of the Siemens AG and the Caché Campus program of the InterSystems GmbH.

**References**

1. Sarnowski C, Sugier PE, Granell R, Jarvis D, Dizier MH, Ege M, et al. Identification of a new locus at 16q12 associated with time to asthma onset. J Allergy Clin Immunol 2016; 138:1071-80.

2. Bulik-Sullivan BK, Loh PR, Finucane HK, Ripke S, Yang J, Schizophrenia Working Group of the Psychiatric Genomics C, et al. LD Score regression distinguishes confounding from polygenicity in genome-wide association studies. Nat Genet 2015; 47:291-5.

3. Moffatt MF, Gut IG, Demenais F, Strachan DP, Bouzigon E, Heath S, et al. A large-scale, consortium-based genomewide association study of asthma. N Engl J Med 2010; 363:1211-21.

4. Demenais F, Margaritte-Jeannin P, Barnes KC, Cookson WOC, Altmuller J, Ang W, et al. Multiancestry association study identifies new asthma risk loci that colocalize with immune-cell enhancer marks. Nat Genet 2018; 50:42-53.

5. Brumpton BM, Langhammer A, Ferreira MA, Chen Y, Mai XM. Physical activity and incident asthma in adults: the HUNT Study, Norway. BMJ Open 2016; 6:e013856.

6. Ahmed S, Maratha A, Butt AQ, Shevlin E, Miggin SM. TRIF-mediated TLR3 and TLR4 signaling is negatively regulated by ADAM15. J Immunol 2013; 190:2217-28.

7. Fourie AM, Coles F, Moreno V, Karlsson L. Catalytic activity of ADAM8, ADAM15, and MDC-L (ADAM28) on synthetic peptide substrates and in ectodomain cleavage of CD23. J Biol Chem 2003; 278:30469-77.

8. Sun C, Beard RS, Jr., McLean DL, Rigor RR, Konia T, Wu MH, et al. ADAM15 deficiency attenuates pulmonary hyperpermeability and acute lung injury in lipopolysaccharide-treated mice. Am J Physiol Lung Cell Mol Physiol 2013; 304:L135-42.

9. Consortium GT, Laboratory DA, Coordinating Center -Analysis Working G, Statistical Methods groups-Analysis Working G, Enhancing Gg, Fund NIHC, et al. Genetic effects on gene expression across human tissues. Nature 2017; 550:204-13.

10. Shimizu E, Yasui A, Matsuura K, Hijiya N, Higuchi Y, Yamamoto S. Structure and expression of the murine ADAM 15 gene and its splice variants, and difference of interaction between their cytoplasmic domains and Src family proteins. Biochem Biophys Res Commun 2003; 309:779-85.

11. Foletta VC. Transcription factor AP-1, and the role of Fra-2. Immunol Cell Biol 1996; 74:121-33.

12. Rani A, Greenlaw R, Runglall M, Jurcevic S, John S. FRA2 is a STAT5 target gene regulated by IL-2 in human CD4 T cells. PLoS One 2014; 9:e90370.

13. Lund RJ, Loytomaki M, Naumanen T, Dixon C, Chen Z, Ahlfors H, et al. Genome-wide identification of novel genes involved in early Th1 and Th2 cell differentiation. J Immunol 2007; 178:3648-60.

14. Ubieta K, Garcia M, Grotsch B, Uebe S, Weber GF, Stein M, et al. Fra-2 regulates B cell development by enhancing IRF4 and Foxo1 transcription. J Exp Med 2017; 214:2059-71.

15. Wurm S, Zhang J, Guinea-Viniegra J, Garcia F, Munoz J, Bakiri L, et al. Terminal epidermal differentiation is regulated by the interaction of Fra-2/AP-1 with Ezh2 and ERK1/2. Genes Dev 2015; 29:144-56.

16. Ciofani M, Madar A, Galan C, Sellars M, Mace K, Pauli F, et al. A validated regulatory network for Th17 cell specification. Cell 2012; 151:289-303.

17. Ye W, Hu MM, Lei CQ, Zhou Q, Lin H, Sun MS, et al. TRIM8 Negatively Regulates TLR3/4-Mediated Innate Immune Response by Blocking TRIF-TBK1 Interaction. J Immunol 2017; 199:1856-64.

18. Rosenzweig BL, Imamura T, Okadome T, Cox GN, Yamashita H, ten Dijke P, et al. Cloning and characterization of a human type II receptor for bone morphogenetic proteins. Proc Natl Acad Sci U S A 1995; 92:7632-6.

19. International PPHC, Lane KB, Machado RD, Pauciulo MW, Thomson JR, Phillips JA, 3rd, et al. Heterozygous germline mutations in BMPR2, encoding a TGF-beta receptor, cause familial primary pulmonary hypertension. Nat Genet 2000; 26:81-4.

20. Rudarakanchana N, Flanagan JA, Chen H, Upton PD, Machado R, Patel D, et al. Functional analysis of bone morphogenetic protein type II receptor mutations underlying primary pulmonary hypertension. Hum Mol Genet 2002; 11:1517-25.

21. Hager-Theodorides AL, Outram SV, Shah DK, Sacedon R, Shrimpton RE, Vicente A, et al. Bone morphogenetic protein 2/4 signaling regulates early thymocyte differentiation. J Immunol 2002; 169:5496-504.

22. Spracklen CN, Chen P, Kim YJ, Wang X, Cai H, Li S, et al. Association analyses of East Asian individuals and trans-ancestry analyses with European individuals reveal new loci associated with cholesterol and triglyceride levels. Hum Mol Genet 2017; 26:1770-84.

23. Fischer A, Ellinghaus D, Nutsua M, Hofmann S, Montgomery CG, Iannuzzi MC, et al. Identification of Immune-Relevant Factors Conferring Sarcoidosis Genetic Risk. Am J Respir Crit Care Med 2015; 192:727-36.

24. Wright GJ, Puklavec MJ, Willis AC, Hoek RM, Sedgwick JD, Brown MH, et al. Lymphoid/neuronal cell surface OX2 glycoprotein recognizes a novel receptor on macrophages implicated in the control of their function. Immunity 2000; 13:233-42.

25. Wright GJ, Cherwinski H, Foster-Cuevas M, Brooke G, Puklavec MJ, Bigler M, et al. Characterization of the CD200 receptor family in mice and humans and their interactions with CD200. J Immunol 2003; 171:3034-46.

26. Hoek RM, Ruuls SR, Murphy CA, Wright GJ, Goddard R, Zurawski SM, et al. Down-regulation of the macrophage lineage through interaction with OX2 (CD200). Science 2000; 290:1768-71.

27. Cherwinski HM, Murphy CA, Joyce BL, Bigler ME, Song YS, Zurawski SM, et al. The CD200 receptor is a novel and potent regulator of murine and human mast cell function. J Immunol 2005; 174:1348-56.

28. Jenmalm MC, Cherwinski H, Bowman EP, Phillips JH, Sedgwick JD. Regulation of myeloid cell function through the CD200 receptor. J Immunol 2006; 176:191-9.

29. Fallarino F, Asselin-Paturel C, Vacca C, Bianchi R, Gizzi S, Fioretti MC, et al. Murine plasmacytoid dendritic cells initiate the immunosuppressive pathway of tryptophan catabolism in response to CD200 receptor engagement. J Immunol 2004; 173:3748-54.

30. Lauzon-Joset JF, Langlois A, Lai LJ, Santerre K, Lee-Gosselin A, Bosse Y, et al. Lung CD200 Receptor Activation Abrogates Airway Hyperresponsiveness in Experimental Asthma. Am J Respir Cell Mol Biol 2015; 53:276-84.

31. Kos O, Hughson RL, Hart DA, Clement G, Frings-Meuthen P, Linnarsson D, et al. Elevated serum soluble CD200 and CD200R as surrogate markers of bone loss under bed rest conditions. Bone 2014; 60:33-40.

32. Ferreira RC, Freitag DF, Cutler AJ, Howson JM, Rainbow DB, Smyth DJ, et al. Functional IL6R 358Ala Allele Impairs Classical IL-6 Receptor Signaling and Influences Risk of Diverse Inflammatory Diseases. PLoS Genet 2013; 9:e1003444.

33. Snelgrove RJ, Goulding J, Didierlaurent AM, Lyonga D, Vekaria S, Edwards L, et al. A critical function for CD200 in lung immune homeostasis and the severity of influenza infection. Nat Immunol 2008; 9:1074-83.

34. Brezar V, Tu WJ, Seddiki N. PKC-Theta in Regulatory and Effector T-cell Functions. Front Immunol 2015; 6:530.

35. Kong KF, Altman A. In and out of the bull's eye: protein kinase Cs in the immunological synapse. Trends Immunol 2013; 34:234-42.

36. Salek-Ardakani S, So T, Halteman BS, Altman A, Croft M. Differential regulation of Th2 and Th1 lung inflammatory responses by protein kinase C theta. J Immunol 2004; 173:6440-7.

37. Madouri F, Chenuet P, Beuraud C, Fauconnier L, Marchiol T, Rouxel N, et al. Protein kinase Ctheta controls type 2 innate lymphoid cell and TH2 responses to house dust mite allergen. J Allergy Clin Immunol 2017; 139:1650-66.

38. Sen S, Wang F, Zhang J, He Z, Ma J, Gwack Y, et al. SRC1 promotes Th17 differentiation by overriding Foxp3 suppression to stimulate RORgammat activity in a PKC-theta-dependent manner. Proc Natl Acad Sci U S A 2018; 115:E458-E67.

39. Gupta S, Manicassamy S, Vasu C, Kumar A, Shang W, Sun Z. Differential requirement of PKC-theta in the development and function of natural regulatory T cells. Mol Immunol 2008; 46:213-24.

40. Ogura Y, Bonen DK, Inohara N, Nicolae DL, Chen FF, Ramos R, et al. A frameshift mutation in NOD2 associated with susceptibility to Crohn's disease. Nature 2001; 411:603-6.

41. Hugot JP, Chamaillard M, Zouali H, Lesage S, Cezard JP, Belaiche J, et al. Association of NOD2 leucine-rich repeat variants with susceptibility to Crohn's disease. Nature 2001; 411:599-603.

42. Huang H, Fang M, Jostins L, Umicevic Mirkov M, Boucher G, Anderson CA, et al. Fine-mapping inflammatory bowel disease loci to single-variant resolution. Nature 2017; 547:173-8.

43. Girardin SE, Boneca IG, Viala J, Chamaillard M, Labigne A, Thomas G, et al. Nod2 is a general sensor of peptidoglycan through muramyl dipeptide (MDP) detection. J Biol Chem 2003; 278:8869-72.

44. Maeda S, Hsu LC, Liu H, Bankston LA, Iimura M, Kagnoff MF, et al. Nod2 mutation in Crohn's disease potentiates NF-kappaB activity and IL-1beta processing. Science 2005; 307:734-8.

45. Netea MG, Kullberg BJ, de Jong DJ, Franke B, Sprong T, Naber TH, et al. NOD2 mediates anti-inflammatory signals induced by TLR2 ligands: implications for Crohn's disease. Eur J Immunol 2004; 34:2052-9.

46. Kobayashi KS, Chamaillard M, Ogura Y, Henegariu O, Inohara N, Nunez G, et al. Nod2-dependent regulation of innate and adaptive immunity in the intestinal tract. Science 2005; 307:731-4.

47. Sabbah A, Chang TH, Harnack R, Frohlich V, Tominaga K, Dube PH, et al. Activation of innate immune antiviral responses by Nod2. Nat Immunol 2009; 10:1073-80.

48. Zhang Y, Feng X, We R, Derynck R. Receptor-associated Mad homologues synergize as effectors of the TGF-beta response. Nature 1996; 383:168-72.

49. Kim BG, Li C, Qiao W, Mamura M, Kasprzak B, Anver M, et al. Smad4 signalling in T cells is required for suppression of gastrointestinal cancer. Nature 2006; 441:1015-9.

50. Hahn JN, Falck VG, Jirik FR. Smad4 deficiency in T cells leads to the Th17-associated development of premalignant gastroduodenal lesions in mice. J Clin Invest 2011; 121:4030-42.

51. Zhang S, Takaku M, Zou L, Gu AD, Chou WC, Zhang G, et al. Reversing SKI-SMAD4-mediated suppression is essential for TH17 cell differentiation. Nature 2017; 551:105-9.

52. Ebel ME, Kansas GS. Functions of Smad Transcription Factors in TGF-beta1-Induced Selectin Ligand Expression on Murine CD4 Th Cells. J Immunol 2016; 197:2627-34.

53. Wang A, Pan D, Lee YH, Martinez GJ, Feng XH, Dong C. Cutting edge: Smad2 and Smad4 regulate TGF-beta-mediated Il9 gene expression via EZH2 displacement. J Immunol 2013; 191:4908-12.

54. Abe-Dohmae S, Ueda K, Yokoyama S. ABCA7, a molecule with unknown function. FEBS Lett 2006; 580:1178-82.

55. Kaminski WE, Orso E, Diederich W, Klucken J, Drobnik W, Schmitz G. Identification of a novel human sterol-sensitive ATP-binding cassette transporter (ABCA7). Biochem Biophys Res Commun 2000; 273:532-8.

56. Jehle AW, Gardai SJ, Li S, Linsel-Nitschke P, Morimoto K, Janssen WJ, et al. ATP-binding cassette transporter A7 enhances phagocytosis of apoptotic cells and associated ERK signaling in macrophages. J Cell Biol 2006; 174:547-56.

57. Nowyhed HN, Chandra S, Kiosses W, Marcovecchio P, Andary F, Zhao M, et al. ATP Binding Cassette Transporter ABCA7 Regulates NKT Cell Development and Function by Controlling CD1d Expression and Lipid Raft Content. Sci Rep 2017; 7:40273.

58. Kielar D, Kaminski WE, Liebisch G, Piehler A, Wenzel JJ, Mohle C, et al. Adenosine triphosphate binding cassette (ABC) transporters are expressed and regulated during terminal keratinocyte differentiation: a potential role for ABCA7 in epidermal lipid reorganization. J Invest Dermatol 2003; 121:465-74.

59. Steinberg S, Stefansson H, Jonsson T, Johannsdottir H, Ingason A, Helgason H, et al. Loss-of-function variants in ABCA7 confer risk of Alzheimer's disease. Nat Genet 2015; 47:445-7.

60. Hollingworth P, Harold D, Sims R, Gerrish A, Lambert JC, Carrasquillo MM, et al. Common variants at ABCA7, MS4A6A/MS4A4E, EPHA1, CD33 and CD2AP are associated with Alzheimer's disease. Nat Genet 2011; 43:429-35.

61. Bednash JS, Mallampalli RK. Regulation of inflammasomes by ubiquitination. Cell Mol Immunol 2016; 13:722-8.

62. Fu B, Li S, Wang L, Berman MA, Dorf ME. The ubiquitin conjugating enzyme UBE2L3 regulates TNFalpha-induced linear ubiquitination. Cell Res 2014; 24:376-9.

63. Lewis MJ, Vyse S, Shields AM, Boeltz S, Gordon PA, Spector TD, et al. UBE2L3 polymorphism amplifies NF-kappaB activation and promotes plasma cell development, linking linear ubiquitination to multiple autoimmune diseases. Am J Hum Genet 2015; 96:221-34.

64. Simmons A, Gangadharan B, Hodges A, Sharrocks K, Prabhakar S, Garcia A, et al. Nef-mediated lipid raft exclusion of UbcH7 inhibits Cbl activity in T cells to positively regulate signaling. Immunity 2005; 23:621-34.

65. Kathania M, Zeng M, Yadav VN, Moghaddam SJ, Yang B, Venuprasad K. Ndfip1 regulates itch ligase activity and airway inflammation via UbcH7. J Immunol 2015; 194:2160-7.

66. Ferreira MA, Vonk JM, Baurecht H, Marenholz I, Tian C, Hoffman JD, et al. Shared genetic origin of asthma, hay fever and eczema elucidates allergic disease biology. Nat Genet 2017; 49:1752-7.

67. Altin JA, Daley SR, Howitt J, Rickards HJ, Batkin AK, Horikawa K, et al. Ndfip1 mediates peripheral tolerance to self and exogenous antigen by inducing cell cycle exit in responding CD4+ T cells. Proc Natl Acad Sci U S A 2014; 111:2067-74.

68. Layman AAK, Sprout SL, Phillips D, Oliver PM. Ndfip1 restricts Th17 cell potency by limiting lineage stability and proinflammatory cytokine production. Sci Rep 2017; 7:39649.

69. Eldridge MJG, Sanchez-Garrido J, Hoben GF, Goddard PJ, Shenoy AR. The Atypical Ubiquitin E2 Conjugase UBE2L3 Is an Indirect Caspase-1 Target and Controls IL-1beta Secretion by Inflammasomes. Cell Rep 2017; 18:1285-97.

70. Boyd A, Golding J, Macleod J, Lawlor DA, Fraser A, Henderson J, et al. Cohort Profile: the 'children of the 90s'--the index offspring of the Avon Longitudinal Study of Parents and Children. Int J Epidemiol. 2013;42(1):111-27.

71 Fraser A, Macdonald-Wallis C, Tilling K, Boyd A, Golding J, Davey Smith G, et al. Cohort Profile: the Avon Longitudinal Study of Parents and Children: ALSPAC mothers cohort. Int J Epidemiol. 2013;42(1):97-110.
