# Supplementary material for: Age-of-onset information helps identify 76 genetic variants associated with allergic disease
Source: PLoS Genet. 2020 Jun 30;16(6):e1008725. doi: 10.1371/journal.pgen.1008725 (PMC7367489; doi:10.1371/journal.pgen.1008725)
Supplement: S1 Fig — (DOCX) [file pgen.1008725.s002.docx]

| 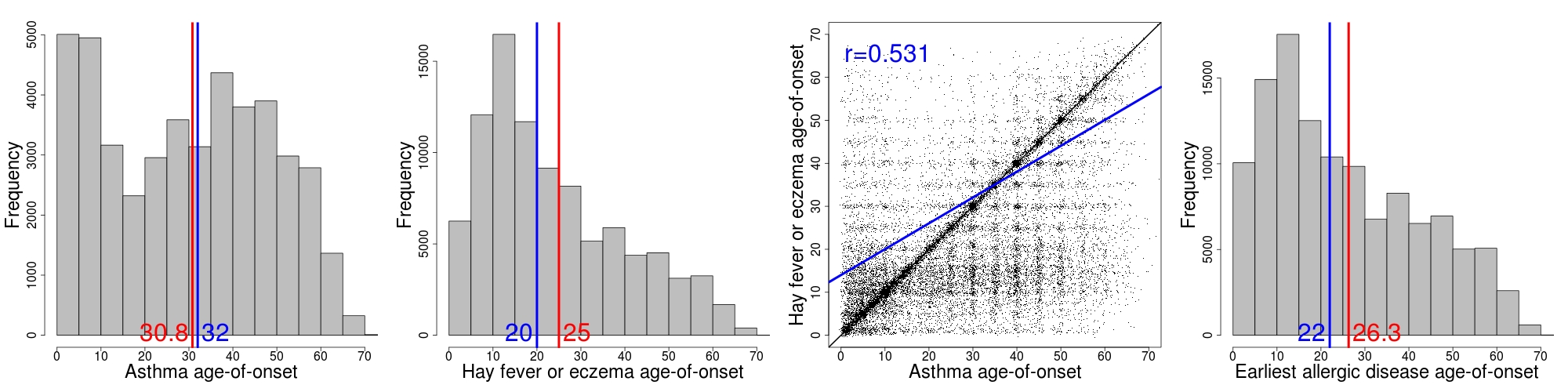 |
| --- |
| **Supplementary Figure 1** |
| Distribution of allergic disease age-of-onset in UK Biobank participants (n=117,130) who reported suffering from asthma and/or hay fever/eczema. |
| Specifically, from left, plot 1 shows age-of-onset for asthma (data from questionnaire item 3786: "What was your age when the asthma was first diagnosed?”). Plot 2 shows age-of-onset of hay fever/eczema (data from questionnaire item 3761: “What was your age when the hayfever, rhinitis or eczema was first diagnosed?”). The mean and median of each distribution are shown in red and blue, respectively. Plot 3 shows the correlation between asthma and hay fever/eczema age-of-onset (to help visualization, a random number from a normal distribution with mean 0 and SD of 0.5 was added to each observation). Plot 4 shows the distribution of the earliest age-of-onset when considering asthma and hay fever/eczema information (this was the variable used in the age-of-onset GWAS described in our study, which was quantile-normalized prior to association analysis). |
