# Supplementary material for: Age-of-onset information helps identify 76 genetic variants associated with allergic disease
Source: PLoS Genet. 2020 Jun 30;16(6):e1008725. doi: 10.1371/journal.pgen.1008725 (PMC7367489; doi:10.1371/journal.pgen.1008725)
Supplement: S2 Fig — (DOCX) [file pgen.1008725.s003.docx]

| 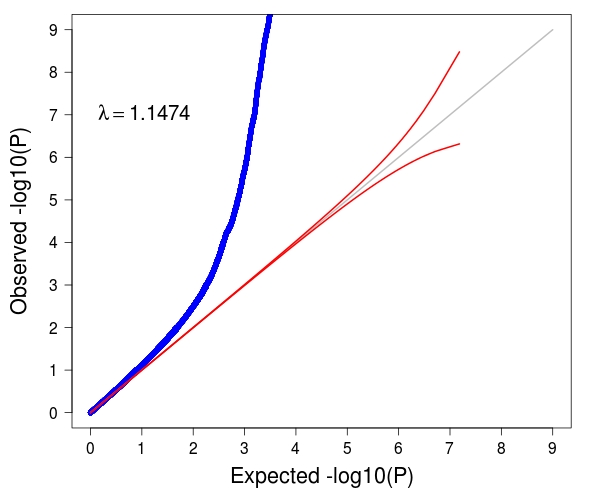 |
| --- |
| **Supplementary Figure 2** |
| Distribution of the observed and expected association P-values for the GWAS of allergic disease age-of-onset in the UK Biobank study (n=117,130). |
| The genomic inflation factor (estimated as the median chi-square divided by 0.4549) was 1.1474, while the LD-score regression intercept was 1.0249. |
