# Supplementary material for: Age-of-onset information helps identify 76 genetic variants associated with allergic disease
Source: PLoS Genet. 2020 Jun 30;16(6):e1008725. doi: 10.1371/journal.pgen.1008725 (PMC7367489; doi:10.1371/journal.pgen.1008725)
Supplement: S3 Fig — (DOCX) [file pgen.1008725.s004.docx]

| 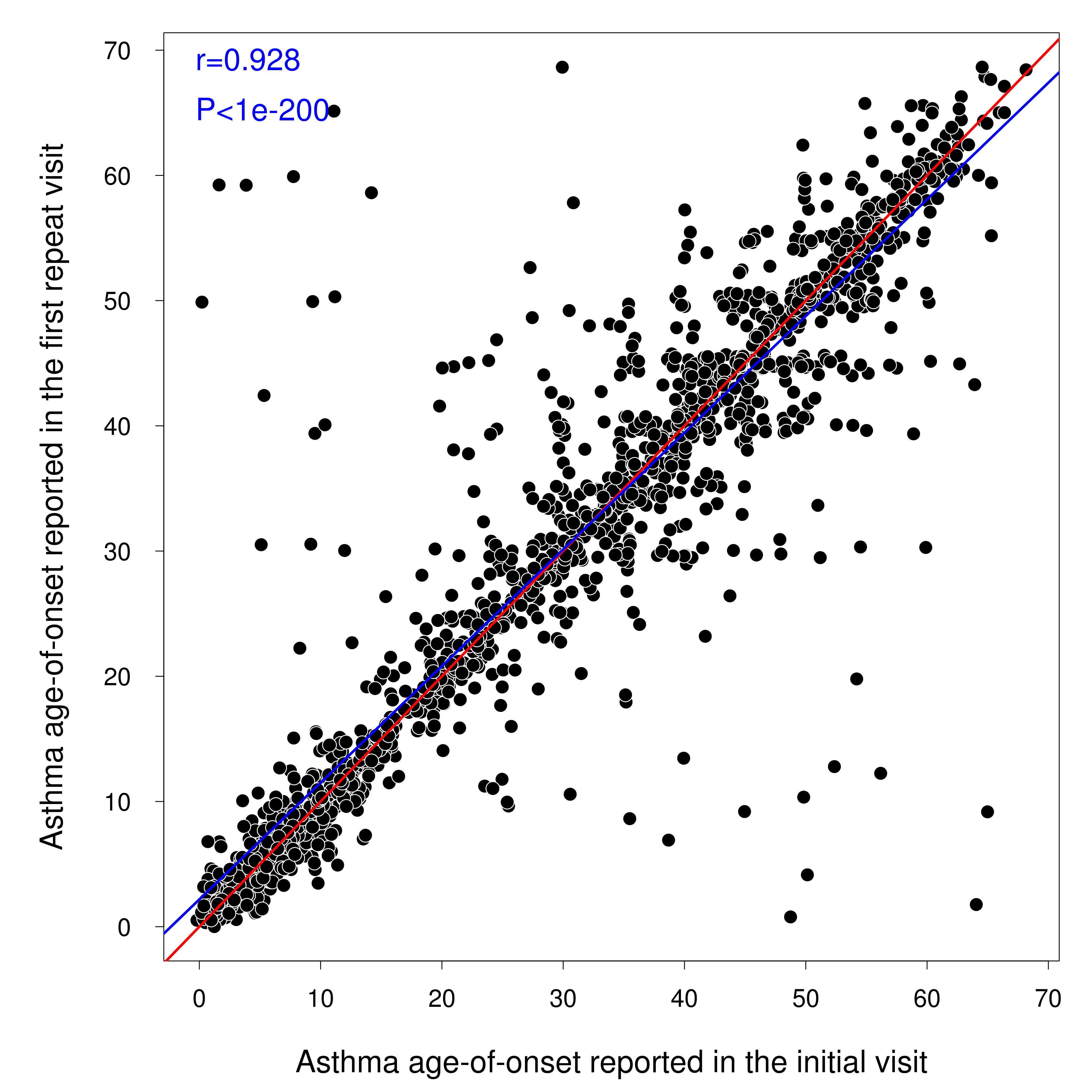 |
| --- |
| **Supplementary Figure** 3 |
| Reliability of self-reported age-of-onset of asthma based on information provided by 1,650 UK Biobank participants at two time points. |
| We compared the reported age at which asthma was diagnosed by a doctor (field 3786: “What was your age when the asthma was first diagnosed?”) between two separate surveys, the initial assessment visit (completed between 2006 and 2010) and the first repeat assessment visit (completed between 2012 and 2013). A total of 1,650 individuals answered the same question at these two time points, with a strong correlation in the reported age-of-onset of asthma between the two (Pearson correlation of 0.93). To help visualization, a random number from a normal distribution with mean 0 and SD of 0.5 was added to each observation in this plot. Lines in red and blue show respectively equality of observations (x=y) and the observed association between the two variables (beta=0.93, intercept=2.2). |
