# Supplementary material for: Age-of-onset information helps identify 76 genetic variants associated with allergic disease
Source: PLoS Genet. 2020 Jun 30;16(6):e1008725. doi: 10.1371/journal.pgen.1008725 (PMC7367489; doi:10.1371/journal.pgen.1008725)
Supplement: S5 Fig — (DOCX) [file pgen.1008725.s006.docx]

| 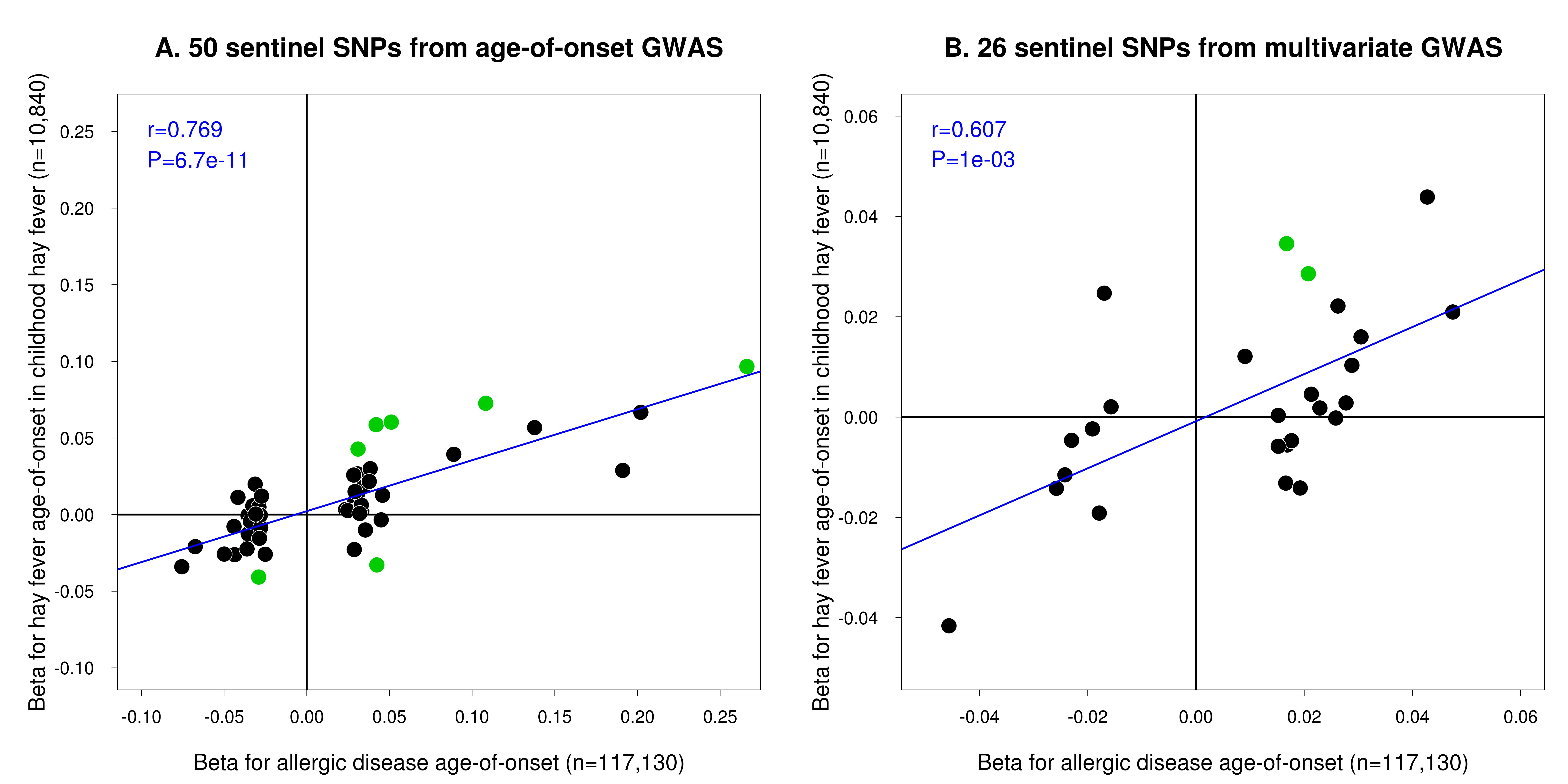 |
| --- |
| **Supplementary Figure 5** |
| Association between sentinel SNPs identified in the age-of-onset (Panel A) or multivariate GWAS (Panel B) and hay fever age-of-onset in the subset of UK Biobank individuals who reported developing hay fever as a child. |
| Comparison of SNP effect (i.e. beta from linear regression) between the analysis of (i) allergic disease age-of-onset (which considers information from asthma, hay fever and eczema onset reported by 117,130 cases from the UK Biobank study; x-axis); and (ii) hay fever age-of-onset in 10,840 individuals from the UK Biobank study who reported developing hay fever as a child (i.e. on or before age 19; y-axis). Panels (A) and (B) show respectively results for the 50 sentinel SNPs identified in the age-of-onset GWAS and the additional 26 sentinel SNPs identified in the multivariate GWAS. SNPs associated with hay fever age-of-onset at a P-value <0.05 are highlighted in green. |
