# Supplementary material for: Age-of-onset information helps identify 76 genetic variants associated with allergic disease
Source: PLoS Genet. 2020 Jun 30;16(6):e1008725. doi: 10.1371/journal.pgen.1008725 (PMC7367489; doi:10.1371/journal.pgen.1008725)
Supplement: S6 Fig — (DOCX) [file pgen.1008725.s007.docx]

| 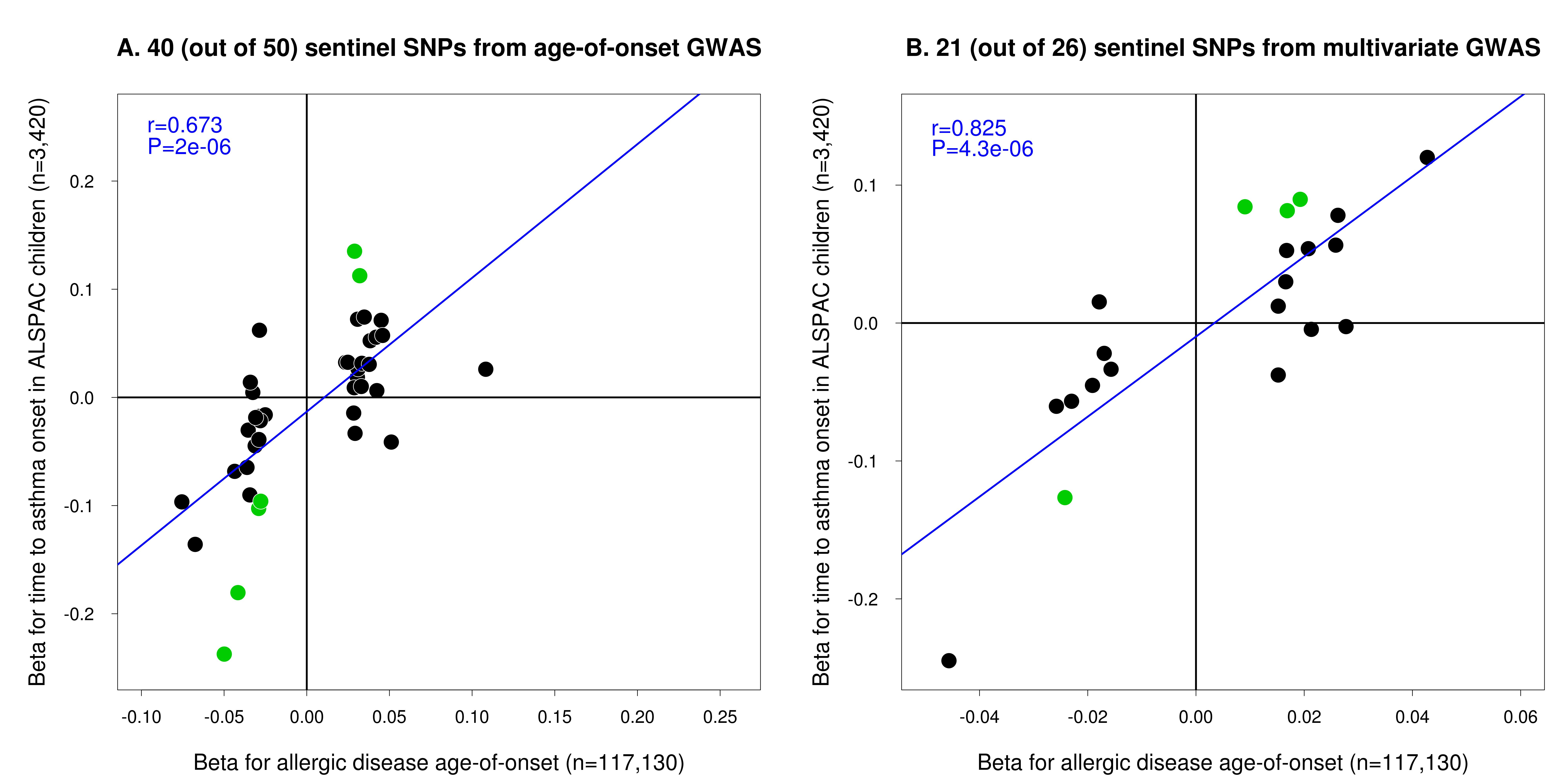 |
| --- |
| **Supplementary Figure 6** |
| Association between sentinel SNPs identified in the age-of-onset (Panel A) or multivariate GWAS (Panel B) and time to asthma onset in children of the ALSPAC study. |
| Comparison of SNP effect (i.e. beta from linear regression) between the analysis of (i) allergic disease age-of-onset (which considers information from asthma, hay fever and eczema onset reported by 117,130 cases from the UK Biobank study; x-axis); and (ii) time to asthma onset in 3,420 children from the ALSPAC study. Panels (A) and (B) show respectively results for 40 (out of 50) sentinel SNPs identified in the age-of-onset GWAS and available in the ALSPAC GWAS, and 21 (out of 26) additional sentinel SNPs identified in the multivariate GWAS and available in the ALSPAC GWAS. SNPs associated with time to asthma onset at a P-value <0.05 are highlighted in green. |
