# Supplementary material for: Age-of-onset information helps identify 76 genetic variants associated with allergic disease
Source: PLoS Genet. 2020 Jun 30;16(6):e1008725. doi: 10.1371/journal.pgen.1008725 (PMC7367489; doi:10.1371/journal.pgen.1008725)
Supplement: S7 Fig — (DOCX) [file pgen.1008725.s008.docx]

| 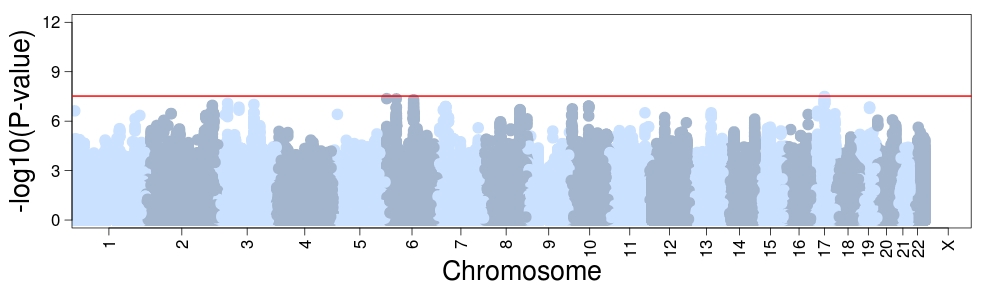 |
| --- |
| **Supplementary Figure 7** |
| Summary of results from the GWAS of allergic disease age-of-onset in the UK Biobank study (n=117,130), after adjusting single-SNP results for the effects of independently associated variants (i.e. with P<3x10^-8^ in the joint association analysis performed with GCTA. |
| No single variant had an association P<3x10^-8^ (red line), as expected. |
