# Supplementary material for: Age-of-onset information helps identify 76 genetic variants associated with allergic disease
Source: PLoS Genet. 2020 Jun 30;16(6):e1008725. doi: 10.1371/journal.pgen.1008725 (PMC7367489; doi:10.1371/journal.pgen.1008725)
Supplement: S8 Fig — (DOCX) [file pgen.1008725.s009.docx]

| 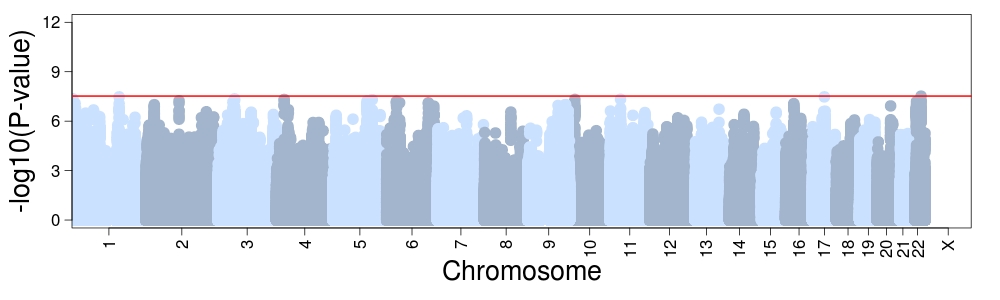 |
| --- |
| **Supplementary Figure 8** |
| Summary of results from the GWAS of allergic disease case-control status (n=360,838) after adjusting single-SNP results for the effects of independently associated variants (i.e. with P<3x10^-8^ in the joint association analysis performed with GCTA). |
| No single variant had an association P<3x10^-8^ (red line), as expected. |
