# Supplementary material for: Age-of-onset information helps identify 76 genetic variants associated with allergic disease
Source: PLoS Genet. 2020 Jun 30;16(6):e1008725. doi: 10.1371/journal.pgen.1008725 (PMC7367489; doi:10.1371/journal.pgen.1008725)
Supplement: S9 Fig — (DOCX) [file pgen.1008725.s010.docx]

| 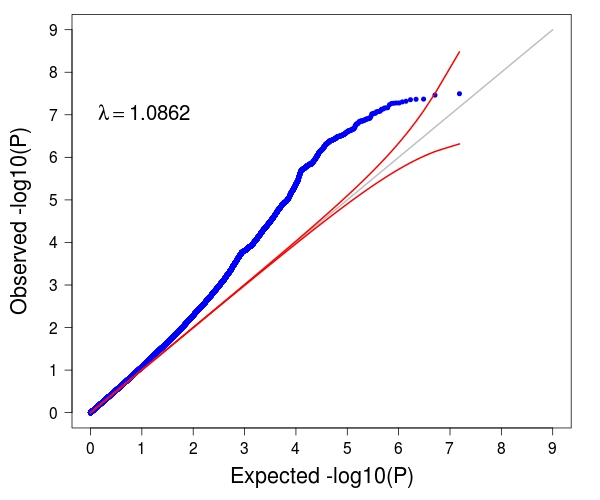 |
| --- |
| **Supplementary Figure 9** |
| Distribution of the observed and expected association P values for the GWAS of allergic disease age-of-onset in the UK Biobank study (n=117,130), after adjusting single-SNP results for the effects of independently associated variants (i.e. with P<3x10^-8^ in the joint association analysis performed with GCTA. |
| The genomic inflation factor (estimated as the median chi-square divided by 0.4549) was 1.0862. |
