# Supplementary material for: Age-of-onset information helps identify 76 genetic variants associated with allergic disease
Source: PLoS Genet. 2020 Jun 30;16(6):e1008725. doi: 10.1371/journal.pgen.1008725 (PMC7367489; doi:10.1371/journal.pgen.1008725)
Supplement: S11 Fig — (DOCX) [file pgen.1008725.s012.docx]

| 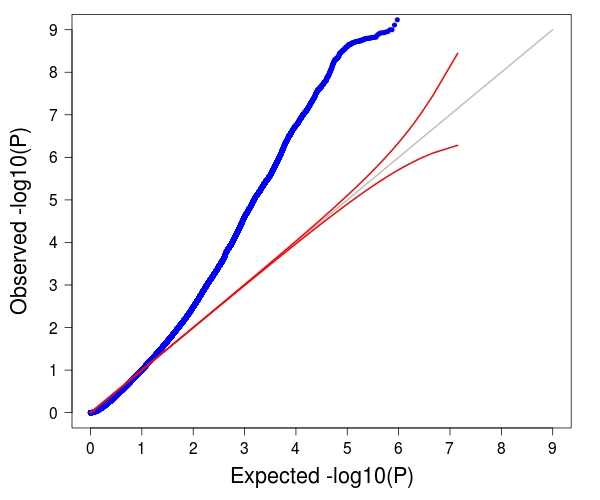 |
| --- |
| **Supplementary Figure 11** |
| Distribution of the observed and expected association P values obtained in the multivariate analysis of the GWAS of allergic disease risk (n=360,838) and GWAS of allergic disease age-of-onset (n=117,130). |
| The multivariate analysis was performed with metaUSAT after adjusting single-SNP results of each GWAS for the effects of independently associated variants. The genomic inflation factor could not be calculated because metaUSAT does not have a closed form null distribution. Nonetheless, inflation of significant associations can be assessed by comparing the observed and expected number of associations significant at a given significance threshold. We observed 38%, 17%, 10%, 5.9% and 1.9% of SNPs tested with a multivariate P-value <0.5, <0.2, <0.1, <0.05 and <0.01, respectively, when the expectations under the null hypothesis of no association were 50%, 20%, 5% and 1%. |
