# Supplementary material for: Age-of-onset information helps identify 76 genetic variants associated with allergic disease
Source: PLoS Genet. 2020 Jun 30;16(6):e1008725. doi: 10.1371/journal.pgen.1008725 (PMC7367489; doi:10.1371/journal.pgen.1008725)
Supplement: S12 Fig — (DOCX) [file pgen.1008725.s013.docx]

| 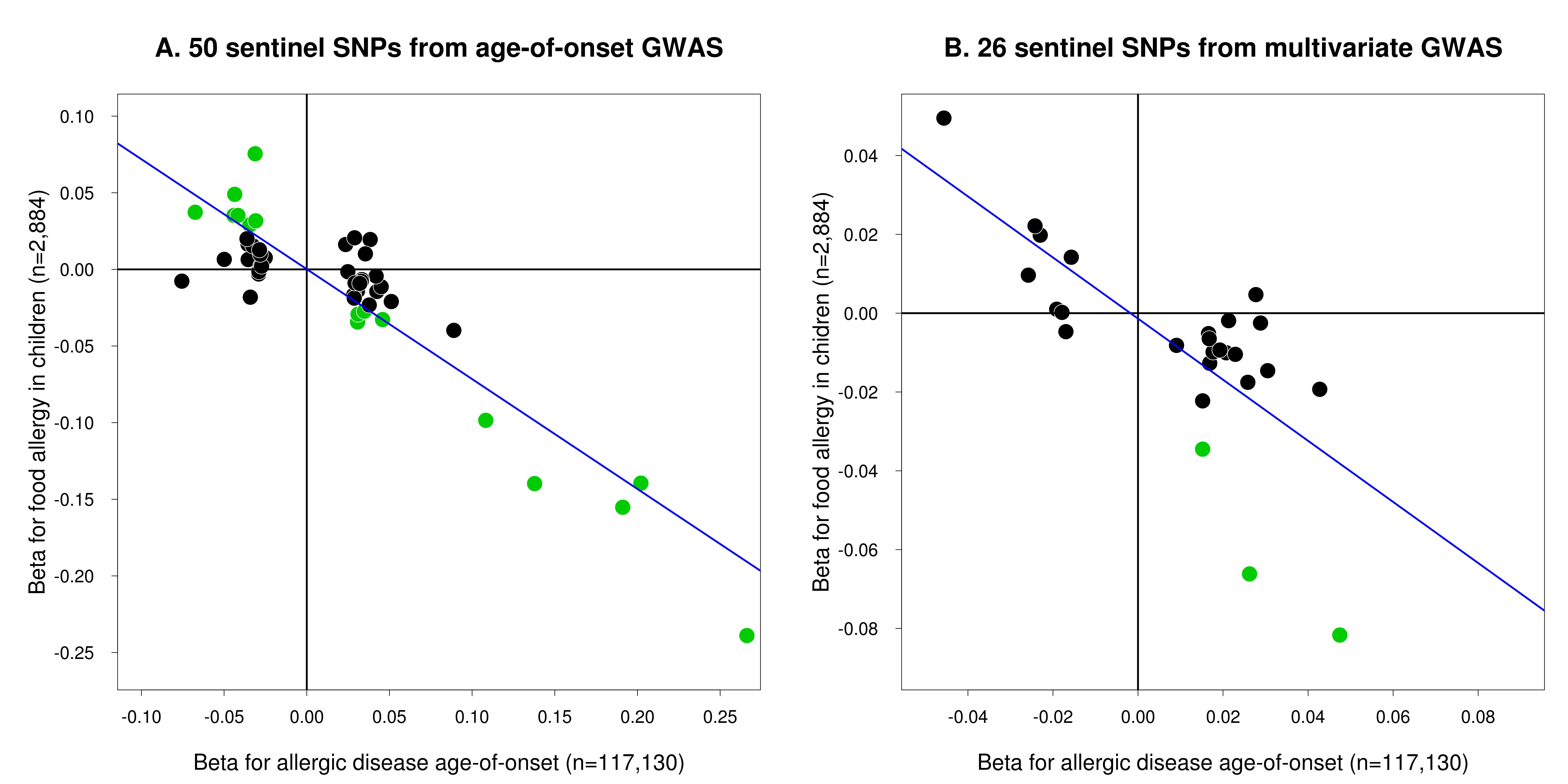 |
| --- |
| **Supplementary Figure 12** |
| Association between sentinel SNPs identified in the age-of-onset (Panel A) or multivariate GWAS (Panel B) and food allergy in children. |
| Comparison of SNP effect (i.e. beta from linear regression) between the analysis of (i) allergic disease age-of-onset (which considers information from asthma, hay fever and eczema onset reported by 117,130 cases from the UK Biobank study; x-axis); and (ii) food allergy case-control status, including 497 children with food allergy diagnosed by oral food challenge in the GOFA study and 2,387 controls (y-axis). Panels (A) and (B) show respectively results for the 50 sentinel SNPs identified in the age-of-onset GWAS and the additional 26 sentinel SNPs identified in the multivariate GWAS. SNPs associated with food allergy at a P-value <0.05 are highlighted in green. |
